# Comprehensive profiling of Asian and Caucasian meibomian gland secretions reveals similar lipidomic signatures regardless of ethnicity

**DOI:** 10.1038/s41598-020-71259-5

**Published:** 2020-09-03

**Authors:** Igor A. Butovich, Tomo Suzuki, Jadwiga Wojtowicz, Nita Bhat, Seher Yuksel

**Affiliations:** 1 Department of Ophthalmology, University of Texas Southwestern Medical Center, 5323 Harry Hines Blvd., Dallas, TX 75390-9057 USA; 2 Graduate School of Biomedical Sciences, University of Texas Southwestern Medical Center, Dallas, TX USA; 3 Department of Ophthalmology, Kyoto Prefectural University of Medicine, Kyoto, Japan; 4 Department of Ophthalmology, Kyoto City Hospital Organization, Kyoto, Japan; 5 Present Address: Centro Oftalmologico de Valencia, Valencia, Venezuela

**Keywords:** Lipidomics, Lipids, Metabolomics, Eye diseases, Eyelid diseases, Analytical biochemistry, Mass spectrometry, Metabolomics, Biochemistry, Biological techniques, Medical research, Risk factors

## Abstract

Meibum—a lipid secretion that is produced by Meibomian glands (MG) in a process termed meibogenesis—plays a critical role in ocular surface physiology. Abnormalities in the chemical composition of meibum were linked to widespread ocular pathologies—dry eye syndrome (DES) and MG dysfunction (MGD). Importantly, in epidemiologic studies the Asian population was shown to be prone to these pathologies more than the Caucasian one, which was tied to differences in their meibomian lipids. However, biochemical data to support these observations and conclusions are limited. To determine if non-DES/non-MGD Asian meibum was significantly different from that of Caucasians, individual samples of meibum collected from ethnic Asian population living in Japan were compared with those of Caucasians living in the USA. These experiments revealed that composition of major lipid classes, such as wax esters (WE), cholesteryl esters (CE), triacylglycerols, *(O)*-acylated ω-hydroxy fatty acids (OAHFA), cholesteryl sulfate, cholesteryl esters of OAHFA, and diacylated α,ω-dihydroxy fatty alcohols remained invariable in both races, barring a minor (< 10%; *p* < 0.01) increase in the Asian CE/WE ratio. Considering the natural variability range for most meibomian lipids (app. ± 15% of the Mean), these differences in meibogenesis were deemed to be minimal and unlikely to have a measurable physiological impact.

## Introduction

Meibomian glands (MG) are holocrine organs that are embedded in the tarsal plates of eyelids of humans and other mammals^1^. The main function of MG is to produce meibum—a lipid secretion that is delivered (either spontaneously or upon blinking) onto the ocular surface through a system of ducts and orifices that open at the rim of the eyelids. Once on the ocular surface, meibum mixes with aqueous tears that are produced by lacrimal glands, to form tear film (TF)^2^. The quality and quantity of meibum are vital for ocular health—TF protects the eye from desiccation, lubricates the eyelids, and maintains proper refractive properties of the cornea, among other functions^3–8^. In vitro studies demonstrated that artificial enrichment of healthy meibum with some of its components that are typically present in vivo in low amounts [such as free cholesterol (Chl), free fatty acids (free FAs), and ceramides], led to dramatic changes in their physicochemical properties and its ability to form stable TF^9,10^. Importantly, several ocular pathologies, such as MG dysfunction (MGD), dry eye syndrome (DES), and chalazia, have been linked to abnormal meibum and TF^11–16^. Meibum characteristics were reported to be dependent on age^17^ and sex^18^, though in our recent studies with human subjects^19,20^ and laboratory animals^21^ no effects of gender on meibomian lipids were observed. Also, our initial report^22^, followed by another communication^23^, questioned the role of ethnicity in meibogenesis. Nevertheless, the role of race in physiology and biochemistry of normal and abnormal MG remains an understudied topic. There are conflicting reports on the role of race/ethnicity in DES and MGD, some of which state that the diseases prevail in Asian population over the Caucasian one^24^, while the others claim the opposite^25^. Notably, much of the evidence that support this observation came from population-wide studies based largely on various questionnaires and routine ophthalmic tests such as Schirmer's tests, measurements of TF break-up time (TFBUT), cornea staining etc*.*. In 2011, Lam et al.^26^ published a very detailed study and reported that meibum of Asian subjects with DES and/or MGD and was quite different from meibum of normal controls. They also suggested that the differences in meibum polar and nonpolar lipid profiles might be responsible for higher prevalence of DES in those groups of the Asian population. Importantly, Lam et al. reported a difference between the overall lipid profiles of Asian and Caucasian meibum. Specifically, Lam et al. found that cholesteryl esters (CE) in Asian meibum represented about 67% of total lipids, while in previous studies with non-Asian population this number had varied between 8 and 39% (Ref.^26^, and references cited therein). Indicatively, in our studies, conducted with predominantly Caucasian volunteers, the family of CE had been estimated to be around 31% of all meibum lipids^27–30^, which is significantly lower than the amount reported by Lam et al. At the same time, wax esters (WE) that accounted for about 41% of the secretion per our data (Refs.^30,31^ and references cited therein) were reported by Lam et al. to constitute about 25% of meibum lipids. Note that no Caucasian volunteers were recruited for the study by Lam et al., and the conclusions were based on chemical analyses of locally collected Asian meibum samples and literature data on the Caucasian cohort, which might not be the optimal design for that study: It would be more appropriate to compare meibum lipids profiles in parallel experiments with samples of different origins, to minimize possible errors that could be introduced by differences in implemented analytical protocols. In a subsequent publication^32^, Lam et al. corrected their initial observations stating improvements in analytical protocols, which brought their new WE estimate to 43% of total lipids in meibum. The new number corresponded well with our earlier report of ~ 40% for WE^33^. However, the level of CE in the study of Lam et al. was still reported to be rather high at 49%. Notably, the new estimates were obtained using tears and meibum collected from DES patients, with no data on normal samples reported, and could not be directly compared with our data on normal subjects. Moreover, all tested subjects were of Asian descent, and a range of major meibomian lipids, such as cholesteryl esters of ω-hydroxy fatty acids (Chl-OAHFA) and α,ω-diacylated diols (DiAD) were not detected and evaluated in either of the studies by Lam et al. Thus, the lingering questions about possible differences between Asian and Caucasian meibum remained unanswered.

These considerations prompted us to conduct a focused study to compare intact Asian and Caucasian meibum in direct, side-by-side experiments using ultra high performance liquid chromatography and high resolution mass spectroscopy (UPLC/MS), which offer high levels of sensitivity and specificity and are the most common analytical tools for targeted and untargeted lipidomic analyses used today. In this paper, we focus solely on the role of ethnicity in meibogenesis, though other factors such as gender^19^, age, hormonal status^34^, diet, climate/weather^35^ etc. *may* be contributing factors to the MG and ocular surface physiology and health. However, including too many variables in one study was deemed impractical because the number of volunteers and study samples that would be needed to satisfy statistical criteria would be increasing in geometric proportion with the number of variables. Therefore, those factors will be evaluated in future projects and reported separately.

## Materials and methods

### Reagents

Authentic WE, CE, and triacylglycerols (TAG) were obtained from Sigma-Aldrich (St. Louis, MO) and Nu-Chek Prep., Inc. (Elysian, MN). HPLC or MS grade organic solvents [iso-propanol (IPA), acetonitrile (AcN), *n*-hexane (Hex), chloroform, methanol (MeOH), water, and > 99% formic and glacial acetic acids (HAc)] were obtained from Sigma-Aldrich, Burdick & Jackson (Muskegon, MI, USA), and Thermo Fisher Scientific (Waltham, MA, USA).

### Study population

The study subjects were recruited at the Kyoto Prefectural University of Medicine and the affiliated Kyoto City Hospital (Japan; KPUM/KCH) and the University of Texas Southwestern Medical Center in Dallas (TX, USA; UTSW). All study sample collection procedures were approved by the Institutional Review Board of UTSW or KPUM/KCH. The procedures were performed in accordance with the tenets set forth in the Declaration of Helsinki. Signed informed consents were obtained from all study participants. Several samples were obtained through the Willed Body Program of UTSW as described earlier^19^. Altogether, 36 samples of Asian and 38 samples of Caucasian meibum that were collected from the same number of individual donors were analyzed (Table S1). The donors underwent regular ocular examination as described before^15,34^. None of the donors had a previous history of MGD, DES, or any other MG-related pathology beyond common age-related minor to mild MG dropout in some elderly donors. No signs of ocular or eyelid inflammation were observed either. As biological sex of the donors has been shown to play no obvious role in meibogenesis in humans and laboratory animals (no differences between the genders on the level of intact individual lipids were observed^19,21^), for the purpose of this study the samples were grouped and analyzed according to the race only. Note, however, that menstrual cycle may somewhat change the FA profiles of meibomian lipids^34^. The effects of this and other factors will be evaluated in future studies.

### Meibum collection and storage

At both locations, samples were collected using surgical microscopes. Strict attention was paid to avoid contamination of the samples by tears and other material from the eyelids. At KPUM/KCH, meibum samples were obtained from the subjects' eyelids by the use of a Daviel cataract spoon and a Yoshitomi MG compressor (T.M.I. Co. Ltd., Saitama, Japan). At UTSW, meibum was expressed using Hardten Eyelid Compression Forceps (Katena Products, Parsippany, NJ, USA), and collected with a platinum microspatula. Meibum specimens were then transferred from the collecting devices into ~ 1 mL of HPLC-grade solvent mixture (CHCl_3_:MeOH = 2:1, v/v) and stored in sealed 2-mL sample glass vials with Teflon-lined lids at −20 °C until further use. Asian samples collected at KPUM/KCH were shipped for analysis to UTSW. Just before shipping, the solvent was evaporated to dryness under a stream of nitrogen, the vials were re-sealed, and the samples were shipped in dry state on ice packs cooled to below −20 °C using overnight delivery service. For subsequent LC/MS experiments, dry samples were re-dissolved in 1 mL of IPA at 35 °C, centrifuged to sediment non-lipid components, and then subjected to LC/MS analysis as is, i.e. without any additional manipulations.

### Chromatography and mass spectrometry of meibum

For screening experiments in the normal phase isocratic HPLC/MS mode, a 717 Plus autosampler, a Waters 1525 binary HPLC pump, a column heater, a temperature control module (all from Waters), and an LCQ Fleet ion trap mass spectrometer with an APCI ion source (both from ThermoFisher) were used. The analyses were conducted as previously described^27,36^. A Diol silica gel column (Lichrosphere 3.2 × 150 mm, 5 μm; from Phenomenex, Torrance, CA) was used. Elution was performed using a Hex:IPA:HAc = 95:5:0.1 (v/v/v) at 35 °C and a 0.2 mL/min flow rate. All analytes were detected in positive ion mode (PIM) in the 100–2,000 Da range. The HPLC/MS data were collected, processed and analyzed using Xcalibur software (from ThermoFisher).

High resolution mass spectrometric analyses were performed on a Waters Synapt G2-Si quadrupole Time-of-Flight (ToF) mass spectrometer with an atmospheric pressure chemical ionization (APCI) IonSabre-II and a low flow ESI ion sources, used interchangeably, and a LockSpray (all from Waters, Milford, MA). The MS analyses were conducted in PIM and negative ion mode (NIM) as described before^19,21^ with a better than 10 mDa accuracy for most analytes (Table S2).

A binary Acquity M-Class ultra-high-performance liquid chromatography (UPLC) system (also from Waters) was used for reverse phase gradient chromatography. All UPLC experiments were conducted on a Acquity UPLC C18 BEH column (1 × 100 mm, 1.7 μm) at 35 °C and 20μL/min flow rate. The analytes were eluted in a binary IPA/AcN gradient with a constant 5% aqueous component that contained 10 mM ammonium formate to facilitate ionization of the analytes in the ion sources exactly as described before^19,21^. A leucine-enkephalin solution was chosen for correcting experimental *m/z* data using the LockSpray option. The experiments were conducted in sensitivity (for quantitation; resolution 10,000 FWHM) and high resolution (for identification; resolution 40,000 FWHM) modes. Three separate datasets were collected for each run—a total ion current chromatogram (TIC), a dataset for MS^E^, and a dataset for the LockSpray corrections. The RP-UPLC/MS data were analyzed using MassLynx (v.4.1) and MS^E^ Data Viewer (v.1.4) (from Waters). The MS^E^ application allowed for automatic alignment of low energy and high energy MS fragmentation data, which facilitated structural characterization and identification of analytes. Before analyzing the data, each run was corrected for background signals by subtracting a blank run (same volume of the vehicle—IPA) using the MassLynx's "Strip" tool.

Gas chromatography–mass spectrometry (GC–MS) analysis of meibum was conducted using a Trace GC Ultra gas chromatograph with a TG-5MS capillary column (30 m × 0.25 mm, film coating 0.25 μm), and an ITQ 1100 ion trap mass spectrometric detector (all from ThermoFisher) exactly as described earlier^33^.

### Untargeted principal component analysis of Asian and Caucasian samples

Meibum is composed of several hundred unique lipid species whose ratio varies depending on physiological conditions of the subjects. Its very nature makes it a logical candidate for analyzing using the Principal Component Analysis (PCA) approach. The Progenesis QI software package (v. 2.4, from Waters/Nonlinear Dynamics) and EZinfo (v. 3.0.3.0 from Waters/Umetrics, Umeå, Sweden) were used to perform PCA analyses. High resolution continuous profile MS data from MassLynx were imported into the Progenesis QI software, analyzed, normalized using total ion counts, and exported to EZinfo for further processing. A loadings biplot was generated using the "Plots and Spreadsheets" routine of EZinfo. The minimal number of components required for adequate representation of the data (Variance explained 98%) was found to be 9 (Supplemental Figure S1).

### Targeted lipidomic analysis of Asian and Caucasian meibum

The PCA approach described in the previous section provided us with a starting point for a more focused targeted LC/MS analysis of Asian and Caucasian meibum. In the past, major meibomian lipids (such as Chl, WE, CE, TAG, phosphatidyl cholines, sphingomyelins, etc.) were quantified using various chromatographic and mass spectrometric techniques^28,33,37–40^. However, many unique meibomian lipids, e.g. Chl-OAHFA and DiAD, cannot be quantitated as no lipid standards exist for them. Also, exact quantitation would require a series of homologous standards as the instrument response often depends on the molecular weights of the analytes^30,33^. Thus, their *relative abundances* (RA) and *partial* RA (PRA) in samples were calculated and compared instead. RA of specific lipids and lipid classes in Asian and Caucasian samples were calculated as the ratio of a sum of all peak areas in extracted ion chromatograms (EIC) of specific ions (Lipid1, Lipid2, … LipidN), and a total ion current measured as a sum of all peaks in corresponding TIC according to Eq. (1):

1 $${\text{Relative Abundance }}\left( {\% {\text{ of total}}} \right) \, = \frac{{100\%  \times { }\sum \left( {{\text{Lipid}}1{ } + {\text{ Lipid}}2{ } + { } \cdots { } + {\text{ LipidN}}} \right)}}{{{\text{Total ion count }}\left( {\text{from integrated TIC in PIM}} \right)}}$$

PRA of individual lipid species within their respective lipid classes were computed for each class of lipid (WE, CE, etc.) using their respective EIC and Eq. (2):

2 $${\text{Partial Relative Abundance }}\left( {\% {\text{ of lipid class}}} \right) \, = \frac{{100\% \times {\text{ Lipid}}1}}{{\sum \left( {{\text{Lipid}}1{ } + {\text{ Lipid}}2{ } + { } \cdots { } + {\text{ LipidN}}} \right)}}$$

Note that RA and PRA of lipids calculated from their experimental MS abundances are not the actual *molar ratios* as the former depend on many factors some of which are mentioned further in the text. However, if experimental conditions are kept constant, then different samples can be compared with each other, and the linear fold changes, or similar parameters, can be calculated for sets of samples, similarly to other disciplines, e.g. genomics. Correspondingly, if no change in RA is detected, than it is reasonable to conclude that the samples are similar, or identical.

### Statistical analyses

The results were analyzed using SigmaStat for Windows v.3.5 (from Systat Software, Inc.). The Kolmogorov–Smirnov Normality tests and Equal Variance tests were performed. The Student's t-test (for normally distributed data) and the Mann–Whitney Rank Sum test (for other situations) were used to compare Asian and Caucasian study groups. Generally, differences in the mean values of two groups are considered to be statistically significant if *p* value is ≤ 0.05. However, when analyzing complex mixtures of many *homologous* compounds (series of WE, CE, TAG, etc.) it is important to realize that sporadic fluctuation in the analytical data for a few members of each group are likely to be observed due to either physiological factors, or experimental and instrument errors, or all of the above. In previous UPLC/MS experiments we established that a standard deviation (SD) for technical replicas of the same sample was about 3.5% of the mean values^19^, while natural variability of *normal* meibum led to SD values ranging from 10 to 25% of the mean values, depending on the type of the analyte and its concentration in the sample^28,29,36,37^. Thus, it seems appropriate to be cautious in claiming physiologically meaningful differences in composition of meibum even if their *p* values are ≤ 0.05.

## Results

### Sample quality control

Meibum samples collected from human donors by digital expression of the secretion from their eyelids are typically small (usually, less than 0.5 mg, even if pooled from two or more eyelids). Moreover, meibum is composed of several hundred lipid species that belong to a large number of different lipid classes, and are present in widely differing molar ratios. The chemical nature of meibum (a mixture of extremely long chain, mostly hydrophobic, lipids) necessitates the use of strong organic solvents for collecting and preparing samples for the analyses. The most common and effective solvents for dissolving lipids are chloroform and its mixtures with other solvents such as methanol, ethanol, *iso*-propanol (IPA) etc.. However, these solvents are also effective in dissolving other compounds, including plastic extractives, skin lipids, skin care products etc. To assure that meibum samples were free of contaminants of that nature, collected samples initially underwent a quality check using normal phase HPLC on a Diol column and ion trap MS in PIM as described in our earlier publications^27,33^ and in the Materials and Methods section. The samples were screened for the presence of typical plastic extractives^36^ such as oleamide (molecular formula C_18_H_35_NO; analytical ion with *m/z* 282.2797), other FA amides, oxidized Irgafos (C_42_H_63_O_4_P, *m/z* 663.4551), di-*iso*-nonyl phthalate (C_26_H_42_O_4_, *m/z* 419.3150) and others. Minor presence of these contaminants in any lipid sample is virtually unavoidable as they exist in some quantities in all commercial organic solvents, but their effects were minimized during post-processing the data as described in Materials and Methods. The samples that contained objectionably high levels of these compounds, which could not be corrected in post-processing (one Asian and one Caucasian samples), were disqualified from further examination.

### Selection of mass spectrometric and chromatographic techniques

Though APCI MS was the method of choice in our previous experiments due to exceptional clarity of the APCI mass spectra (in PIM, most of the analytes produced only proton (M + H)^+^ adducts with no discernible formation of ammonium, sodium and potassium ones), for the purpose of this study we used both APCI and ESI techniques. The latter was included in our protocols in attempt to directly compare our results with those published by Lam et al.^26^, who used exclusively ESI to analyze meibum of Asian patients. For lipid separation, we used a RP-UPLC protocol that had been described in our recent publications^31,34^ and resulted in good separation of individual lipid species within multiple lipid classes, and was compatible with ESI and APCI protocols. The chloroform–methanol eluent utilized by Shui et al.^41^ and Lam et al.^26^ was incompatible with our instrumentation, and is much more toxic.

### ***Characterization of lipids using high resolution mass spectrometry in MS1 and MS***^***E***^*** modes***

**Figure 1:**
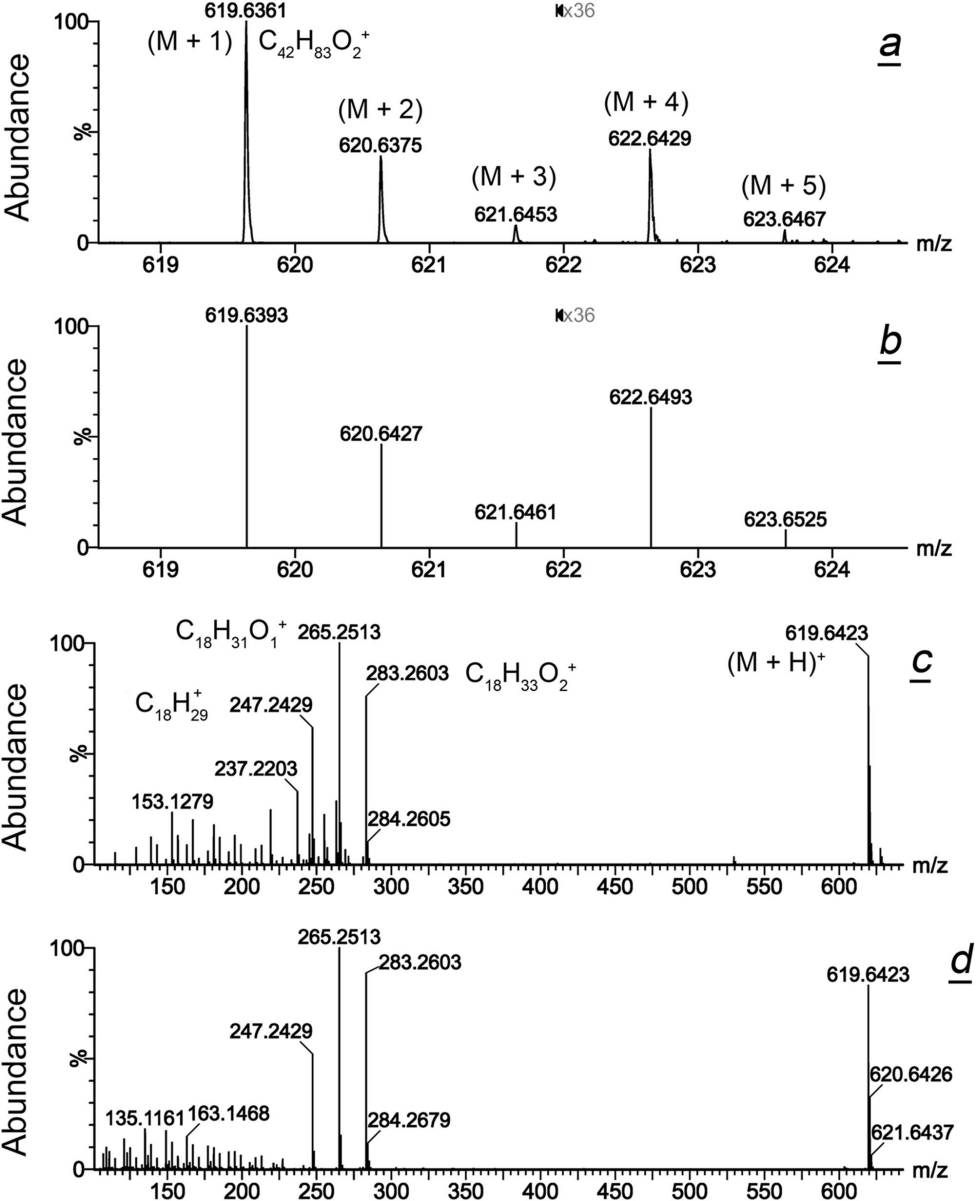
High resolution mass spectrometric analysis of human meibomian lipids using MS and MS^E^ approaches. (**a**) Experimental mass spectrum of lignoceryl oleate; five isotopomers are shown; area between *m/z* 622 and 625 is magnified 36x. (**b**) Theoretical mass spectrum of lignoceryl oleate calculated using the isotope modeling utility of the MasLynx software package. (**c**) MS^E^ fragmentation spectrum of meibomian lignoceryl oleate; (**d**) MS^E^ fragmentation spectrum of authentic lignoceryl oleate.

In our previous studies^28–31,33,36,37,42–46^, we already conducted comprehensive structural characterization and quantitation of major human Meibomian lipids using HPLC/multistage ion trap APCI MS^n^, which made duplication of those experiments in this project unnecessary. Instead, for analyte identification in our current project we relied primarily on the high resolution MS spectra (~ 40,000 FWHM; Fig. 1a,b), while the automatic MS^E^ function, which is available in the Synapt/MassLynx combo, was used as a supplemental approach (Fig. 1c,d). As an example, the first two Fig. 1a,b demonstrate an experimental (1a) and theoretical (1b) mass spectra of a meibomian wax ester with a molecular formula C_42_H_82_O_2_ (lignoceryl oleate) which produced a proton adduct (M + H)^+^ with a theoretical *m/z* 619.6393. Note that the portions of both spectra between *m/z* 622 and 625 were magnified 36x  to show the forth and the fifth isotope peaks of the compound. One can see that a cluster of at least five isotopomers (M + 1) through (M + 5) is detectable and can be used for identification purposes. The wax is available as a pure chemical standard, which allowed us to compare the MS^E^ fragmentation pattern of the natural compound with its synthetic counterpart (Fig. 1c,d). Both compounds fragmented identically to produce a series of ions with *m/z* 283.2603 (protonated oleic acid), *m/z* 265.2513 (oleic acid − H_2_O + H^+^), and *m/z* 247.2429 (oleic acid − 2H_2_O + H^+^). This experiment, together with identical chromatographic retention times of both analytes (not shown), demonstrated that: (1) the major form of the natural wax C_42_H_82_O_2_ was indeed based on oleic acid, and (2) MS^E^ could be used for verification of structures of compounds, if needed.

### Direct gross inter-group comparison of Asian and Caucasian meibum

Next, Asian and Caucasian meibum samples were compared using RP-UPLC/ESI MS in positive ion mode. For each study group, individual runs were combined into a single averaged output data file (ADF) using the "Combine All Files" routine and "Mean Peak Intensities" setting of the MassLynx software—ADF-Asians and ADF-Caucasians (ADF-A and ADF-C), —and analyzed as described below.

**Figure 2:**
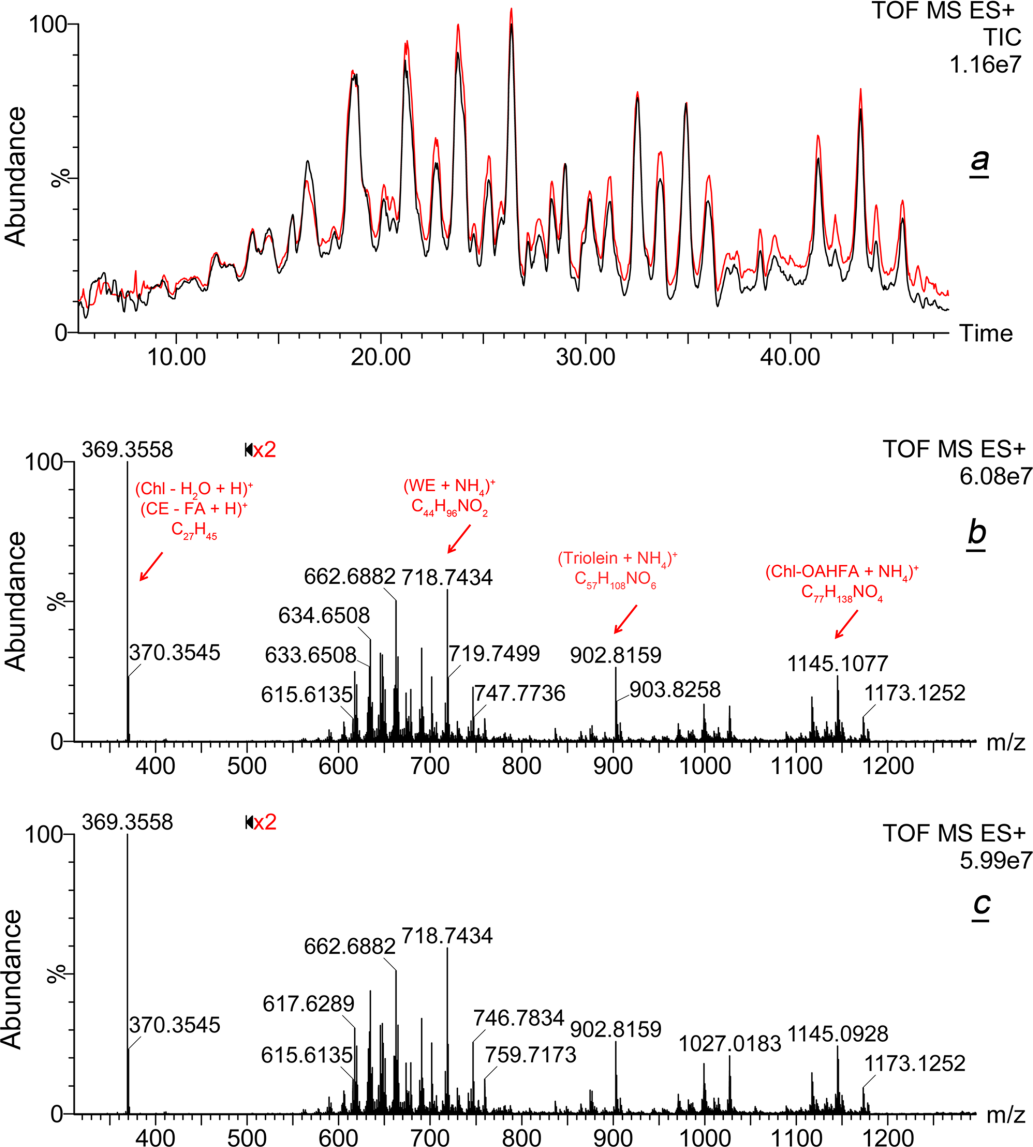
Gross inter-group comparison of Asian and Caucasian meibum. (**a**) RP-UPLC/ESI PIM total ion current chromatograms of Asian (ADF-A, red) and Caucasian (ADF-C, black) meibum. (**b**) Observation mass spectrum of Asian meibum. (**c**) Observation mass spectrum of Caucasian meibum.

A side-by-side, "fingerprint" style comparison of ESI TIC generated from ADF-A and ADF-C data files produced the first clear evidence of their overall similarity: both types of samples replicated each other to the minute details (Fig. 2a). Importantly, the averaged total lipid content in the samples, estimated from their integrated TIC, was high and quite comparable in both groups, with a total ion current for ADF-A and ADF-C being (1.08 ± 0.02) × 10^7^. This fact facilitated their direct side-by-side comparison. Then, observation high resolution ESI mass spectra for both types of samples were obtained from their ADF files (Fig. 2b,c). Notably, averaged mass spectra of Asian and Caucasian meibum were also found to be exceptionally similar with virtually the same ion patterns and signal intensities in both groups. Note that the portions of spectra from *m/z* 500 to 1,300 were magnified in post-processing 2x  to compensate for the lower signal intensity of ions in that area compared to the major ion *m/z* 369.3558.

Most of meibomian lipids were characterized structurally and quantitatively in our previous studies^28–31,33,36,37,42–46^ and related publications by independent groups^26,39,47,48^. Therefore, those experiments will not be described here. However, the structures of major lipid analytes relevant to this manuscript were verified in automatic MS^E^ fragmentation experiments^49,50^ which were a part of every LC/MS analysis conducted in this study. A list of major lipids that were observed in these experiments (Fig. 2b,c) is presented in Table S2. Those included Chl, CE, WE, TAG, OAHFA, DiAD and Chl-OAHFA, which will be discussed in more detail later in the manuscript. As in our previous publications^27,37^, only exceedingly minor (regularly below 0.1% of total meibum), and randomly varying, amounts of phospholipids and sphingomyelins were detected in the current study. Because of their low abundance, they will not be discussed in this paper.

**Figure 3:**
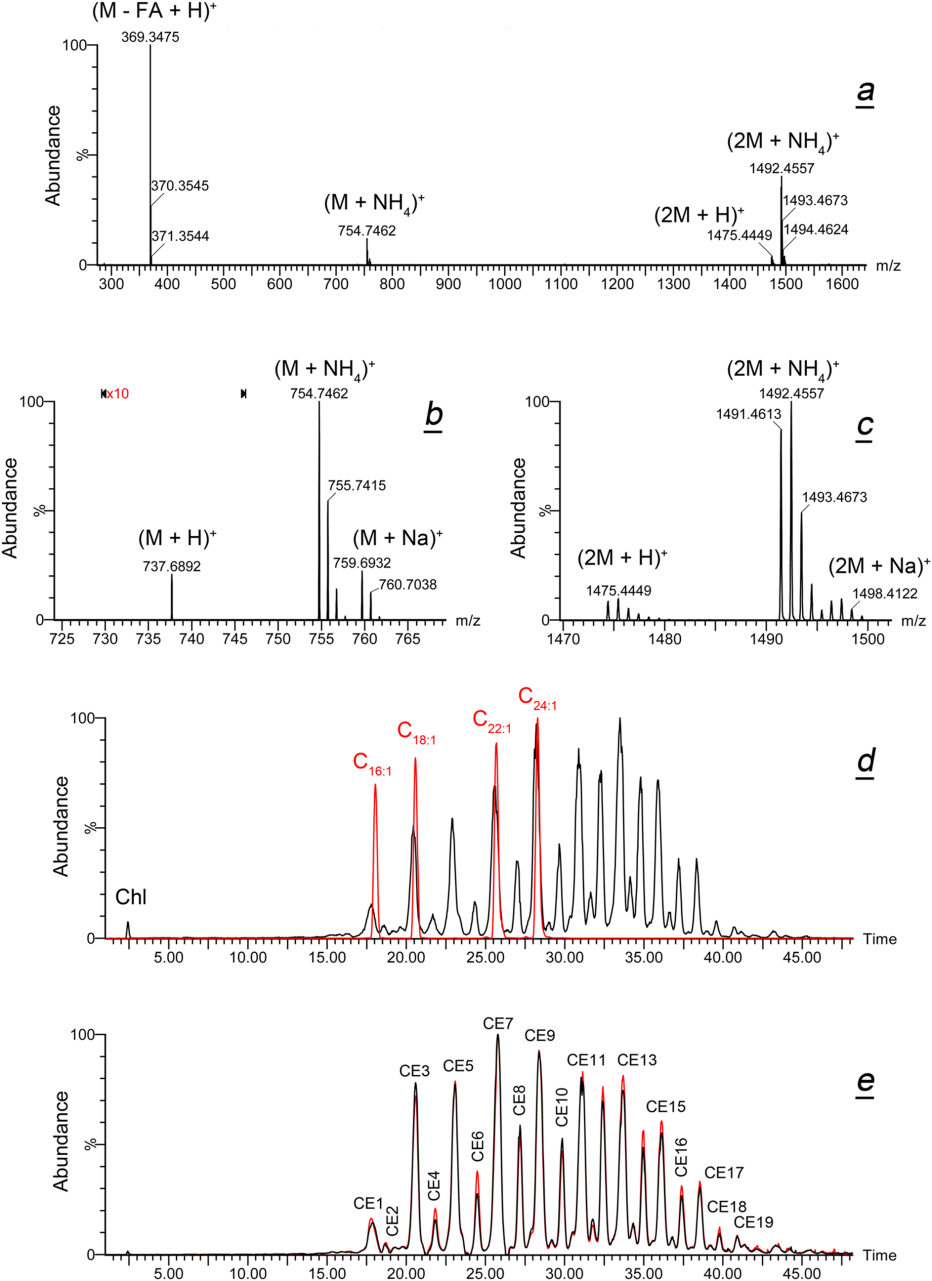
Gross characterization of the cholesteryl ester pools in Asian and Caucasian meibum. (**a**–**c**) High resolution ESI PIM mass spectra of authentic cholesteryl lignocerate demonstrate formation of various adducts of the compound. (**d**) Superposition of extracted ion chromatograms of characteristic ion *m/z* 369.35 obtained for a 50 μM equimolar mixture of four authentic monounsaturated cholesteryl esters (red trace; authentic cholesteryl esters of palmitoleic C_16:1_, oleic C_18:1_, erucic C_22:1_-, and nervonic C_24:1_ fatty acids), with that of a representative human meibum sample (black trace). (**e**) Superposition Asian (ADF-A, red trace) and Caucasian (ADF-C, black trace) chromatograms of characteristic ion *m/z* 369.35 revealed their alikeness.

Then, the CE fractions of both study groups were compared. In the conditions of ESI analysis, authentic CE produced a complex MS which was dominated by their common fragment (M – FA + H)^+^ and CE adducts of the (2M + NH_4_)^+^ nature. Also noticeable were ions (M + NH_4_)^+^, (M + Na)^+^, (2M + H)^+^, and (2M + Na)^+^, while (M + H)^+^ was the weakest of all, which hampered its use in CE identification and quantitation. Representative spectra of authentic cholesteryl lignocerate are shown in Fig. 3a–c. Other individual CE produced equally complex spectra. The strongest analytical ion of all was (M – FA + H)^+^. This common analytical ion with a theoretical *m/z* value of 369.3521 (C_27_H_45_; a proton adduct of dehydro-Chl) is formed from any CE due to its spontaneous fragmentation and a loss of a FA residue in the ion source of the mass spectrometer^27–29,44^. When the EIC of an equimolar 50 μM mixture of four authentic CE with C_16:1_, C_18:1_, C_22:1_, and C_24:1_ FA residues was overlaid with that of human meibum (Fig. 3d), the peaks of standards (red trace) clearly identified their natural counterparts in human meibum (black trace). The latter conclusion was verified by high resolution MS and MS^E^ of individual lipids, as illustrated in Fig. 1 for WE. Note the high level of reproducibility of the analyses in our RP-UPLC/MS experiments^19^.

This approach was used to compare samples collected from humans of different ethnicities. Two ion chromatograms extracted from ADFs of Asian and Caucasian meibum (Fig. 3e) were virtually indistinguishable and demonstrated no significant differences between two ethnicities. Conveniently, the overall presence of CE in the samples, estimated from the integrated EIC of ion *m/z* 369.3521, was almost identical for both groups: the average total ion current for their (M – FA + H)^+^ ions was (7.08 ± 0.10) × 10^6^. Only minor (≤ 9%) variations in the ratios of individual peaks were observed, which were well within a routinely observed variability in individual lipids in human meibum (± 15%, regardless of the study group^19,29,51,52^). Free Chl was virtually undetectable in these experiments in both types of meibum, though it was clearly observable in our earlier and current APCI experiments (see below) as a compound with an RT of about 6.5 ± 0.3 min. Notably, Chl was not detected in the ESI experiments by Lam et al.^26^ either. It seems that poor ionizability of Chl is one of the limitations of the ESI approach for meibum studies. A more detailed evaluation of meibomian CE was conducted using APCI MS and will be discussed later in the text.

Taken together, we found no evidence of noteworthy differences between CE of Asian and Caucasian meibum in RP-UPLC/ESI MS experiments: the lipids were qualitative and quantitatively the same in two ethnicities.

**Figure 4:**
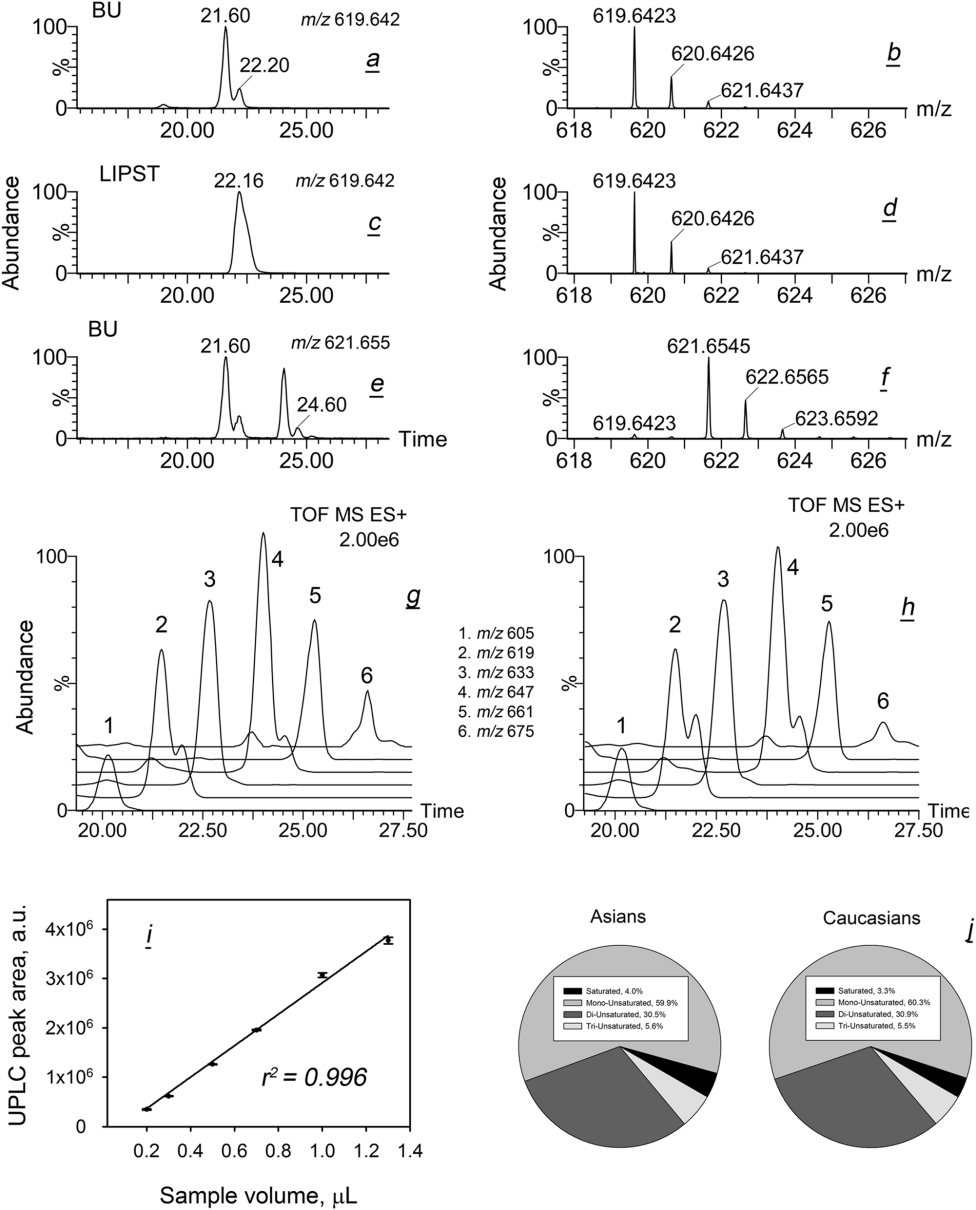
Gross characterization of meibomian wax esters in Asian and Caucasian population. (***a***) RP-UPLC/ESI PIM analyses revealed that at least two isobaric form of lignoceryl oleate with different retention times co-exist in human meibum; extracted ion chromatogram of ion *m/z* 619.64 is shown. (**b**) High resolution mass spectrum of meibomian lignoceryl oleate. (**c**) RP-UPLC analysis of authentic straight chain lignoceryl oleate produced a single UPLC peak with a retention time that matched that of a slower peak from panel (**a**). (**d**) Mass spectrum of authentic lignoceryl oleate. (**e**) Extracted ion chromatogram of ion *m/z* 621.66 observed in human meibum produced four UPLC peaks, two of which were produced by (M + 2) isotopomers of lignoceryl oleate (retention times 21.60 min and 22.20 min), while the last two were produced by proton adducts of lignoceryl stearate; the peak 24.60 min co-eluted with authentic straight chain lignoceryl stearate (not shown). (**f**) Mass spectrum of meibomian lignoceryl oleate matched that of authentic compound. (**g**,**h**) Extracted ion chromatograms of six major wax esters of Asian (ADF-A, panel **g**) and Caucasian (ADF-C, panel **h**) meibum. (**i**) Linearity of the instrument response was verified for major analytes, such as lignoceryl oleate (shown). (**j**) Overall comparison of wax ester pools of Asian and Caucasian meibum revealed their high similarity.

Then, meibomian WE, listed in Table S2, were evaluated. As an example, ESI MS data for lignoceryl oleate (monoisotopic MW 618.6311) and lignoceryl stearate (MW 620.6467) are illustrated in Fig. 4a–f. As a general rule, WE formed (M + H)^+^, (M + NH_4_)^+^ and (M + Na)^+^ adducts, whose relative balance was influenced by the experimental conditions. However, the intensity of WE proton adducts was sufficiently high for reliable detection of all analytes, if LC/MS conditions were kept unchanged. One can see that EIC of lignoceryl oleate produced at least two closely eluting chromatographic peaks with identical MS spectra, which indicated that there were at least two isobaric forms of the compound. Importantly, the slower eluting form (Fig. 4a, peak with RT of 22.20 min) co-eluted with authentic lignoceryl oleate (Fig. 4c, peak with RT of 22.16 min), while the major form of meibomian wax eluted faster and had a RT of 21.60 min. However, MS1 and MS^E^ mass spectra of both forms were indistinguishable. The same conclusions were made for lignoceryl stearate (Fig. 4e,f). The first major UPLC peak with RT of 21.60 was produced by lignoceryl oleate [its third isotope peak has a theoretical *m/z* value of 621.6461 (Fig. 1a,b)], while the second double peak with RT of 24.1/24.60 min was true lignoceryl stearate whose (M+H)^+^ peak has a theoretical *m/z* of 621.6545. The reason for the presence of at least two isobaric forms of each WE is the existence in meibum branched WE^33^. Representative results for six major oleic-acid based meibomian WE—C_41_H_81_O_2_ (proton adduct; theoretical *m/z* 605.6233, trace 1), C_42_H_83_O_2_ (619.6389, trace 2), C_43_H_85_O_2_ (633.6545, trace 3), C_44_H_87_O_2_ (647.6702, trace 4), C_45_H_88_O_2_ (661.6858, trace 5), and C_46_H_90_O_2_ (675.7015, trace 6) are shown in Fig. 4g,h as EIC. Importantly, the instrument response rose linearly with the increase in the amount of injected sample (Fig. 4i).

Other major WE were analyzed in a similar fashion: PRA of major analytes of interest were calculated by integrating corresponding EIC of their (M + H)^+^ adducts. The data on four major classes of WE (saturated and mono-, di-, and tri-unsaturated ones) are summarized in Fig. 4j. Note that Lam et al.^26^ did not report any saturated WE in Asian samples. However, the latter lipids were identified and quantitated using high temperature GC/MS^n^^33^, and were also detectable in all types of samples in the current study. Our current data clearly demonstrated that the overall balance of various types of WE was almost identical in both races.

**Figure 5:**
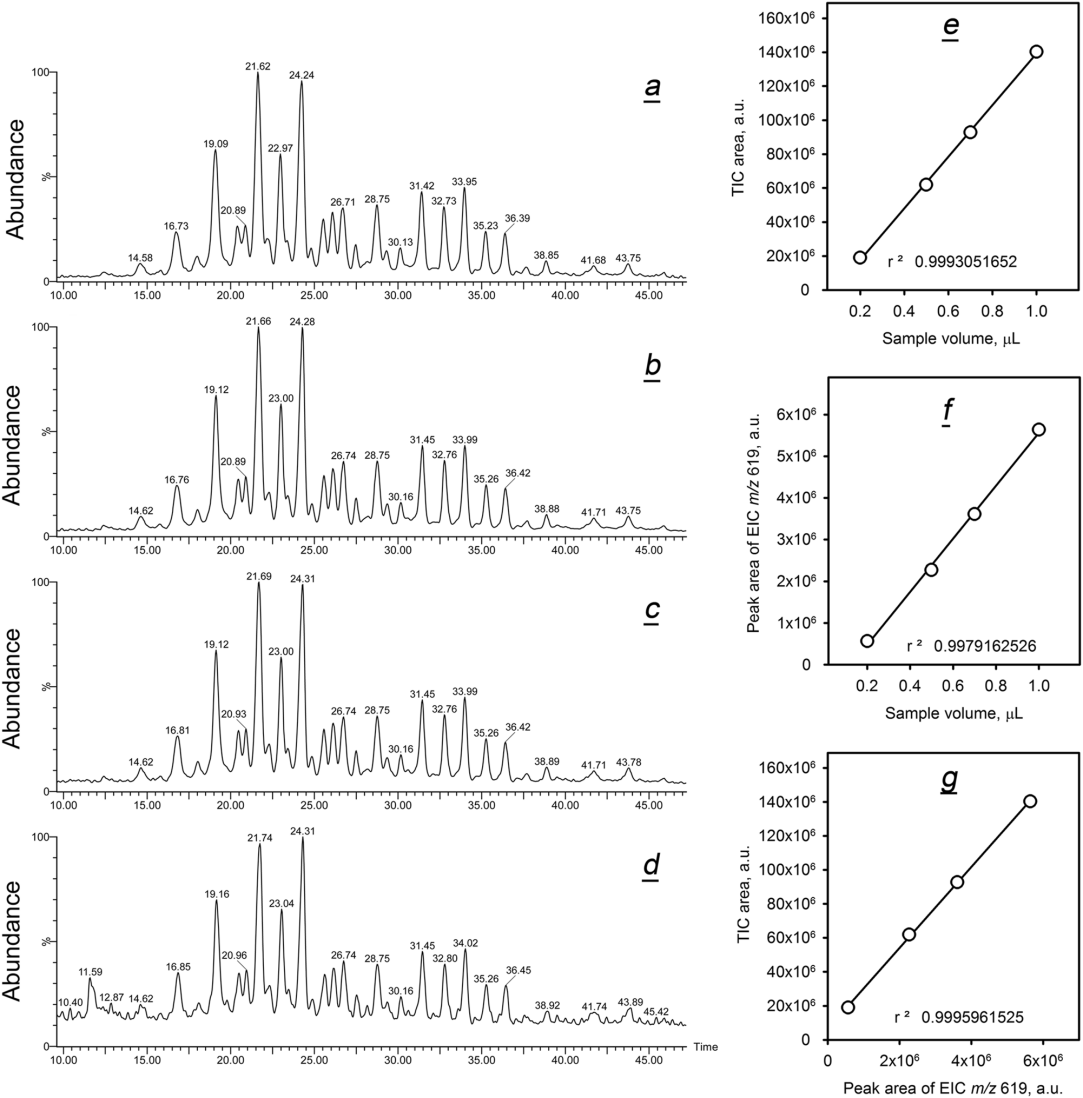
Total ion current chromatograms (TIC) obtained in identical conditions can be used for normalization of meibum samples. (**a–d**) TIC produced by sequential injections of 0.2 μL, 0.5 μL, 0.7 μL, and 1.0 μL of a representative sample human meibum differed only in the signal-to-noise ratio. (**e**) TIC peak areas rose linearly with the amount of the injected sample. (**f**) Peak areas of lignoceryl oleate, measured as extracted ion chromatograms (EIC) of the ion *m/z* 619.64, rose linearly with the amount of the injected sample. (**g**) A linear correlation between TIC and EIC justified the use of TIC as common denominators for comparing different samples.

Finally, we compared the apparent balances of CE and WE in Asian and Caucasian meibum using ADF-A and ADF-C data files. Our starting assumption was that the total ion current for a particular analyte in a sample was a (quasi)linear function of the total ion current of all analytes in the sample. To verify it, the following approach was used. Firstly, the effect of increasing amounts of injected meibum sample on the total ion current measured by the detector was measured by integrating their TIC (Fig. 5a–d). Undeniably, the normalized peak areas and RT of major components were not altered, while absolute TIC rose linearly (*r*^*2*^ > 0.999) with the increase in the amount of the injected sample (Fig. 5e). Note that a typical meibum sample in our study was within the range of TIC peak areas shown in the graph. Secondly, EIC of meibomian wax lignoceryl oleate were generated from those TIC, integrated and corresponding peak areas were plotted as shown in Fig. 5f. Again, a (quasi)linear detector response with *r*^*2*^ = 0.998 was observed. Thirdly, plotting TIC peak areas vs. EIC peak areas demonstrated a perfectly linear relationship between the two (Fig. 5g). These tests were repeated for different combinations of samples and analytes (such as WE, CE, and TAG) with the same outcome, demonstrating that no matrix effects affected the data, and the instrument response was (quasi)linear regardless of the measured parameters. It appeared that TIC could be used as common denominators for comparing RA and PRA of lipids that were measured using their EIC.

Using this approach, RA of CE in Asian and Caucasian samples, measured as the ratio of a sum of all peak areas in EIC of ion *m/z* 369.3517 (CE1, CE2, … CEn) from Fig. 3e, and a total ion current measured as a sum of all peaks in corresponding TIC (such as in Fig. 2a), according to Eq. (1). For Asian meibum, RA of CE was 6.6%, while for Caucasian—an almost identical 6.5%.

Similarly, RA of major WE were calculated. As WE had no common analytical ion, their individual *m/z* values of their (M + H)^+^ adducts were summed instead. Their total RA for Asian and Caucasian meibum were 6.7% and 6.9%, correspondingly.

Thus, the almost identical distribution of CE and WE in both types of meibum led us to a conclusion that the pools of CE and WE in Asian and Caucasian meibomian gland secretions were essentially the same. Minor differences were well within the limits of natural variability of meibomian lipids reported in our previous studies^19,29,51,52^, and experimental errors, and are unlikely to be of physiological significance.

### Intra-group variability of Asian and Caucasian meibum lipid profiles

The experiments discussed in the preceding section provided strong evidence of the overall biochemical similarity of Asian and Caucasian meibum. However, the very nature of such an integrative approach that averaged the data for all samples and analytes for each group made it difficult to visualize and estimate possible *intra*-group differences in the Asian and the Caucasian populations. To gather information on the degree of intra-group variability of meibomian lipidomes, we conducted targeted (or "supervised") lipidomic analysis of individual meibum samples using RP-UPLC/APCI-MS and ESI–MS.

Elution profiles of major individual CE, WE, Chl-OAHFA, TAG, DiAD and OAHFA were obtained for every study sample. The choice of compounds for evaluation was based on our previous publications on the topic.

**Figure 6:**
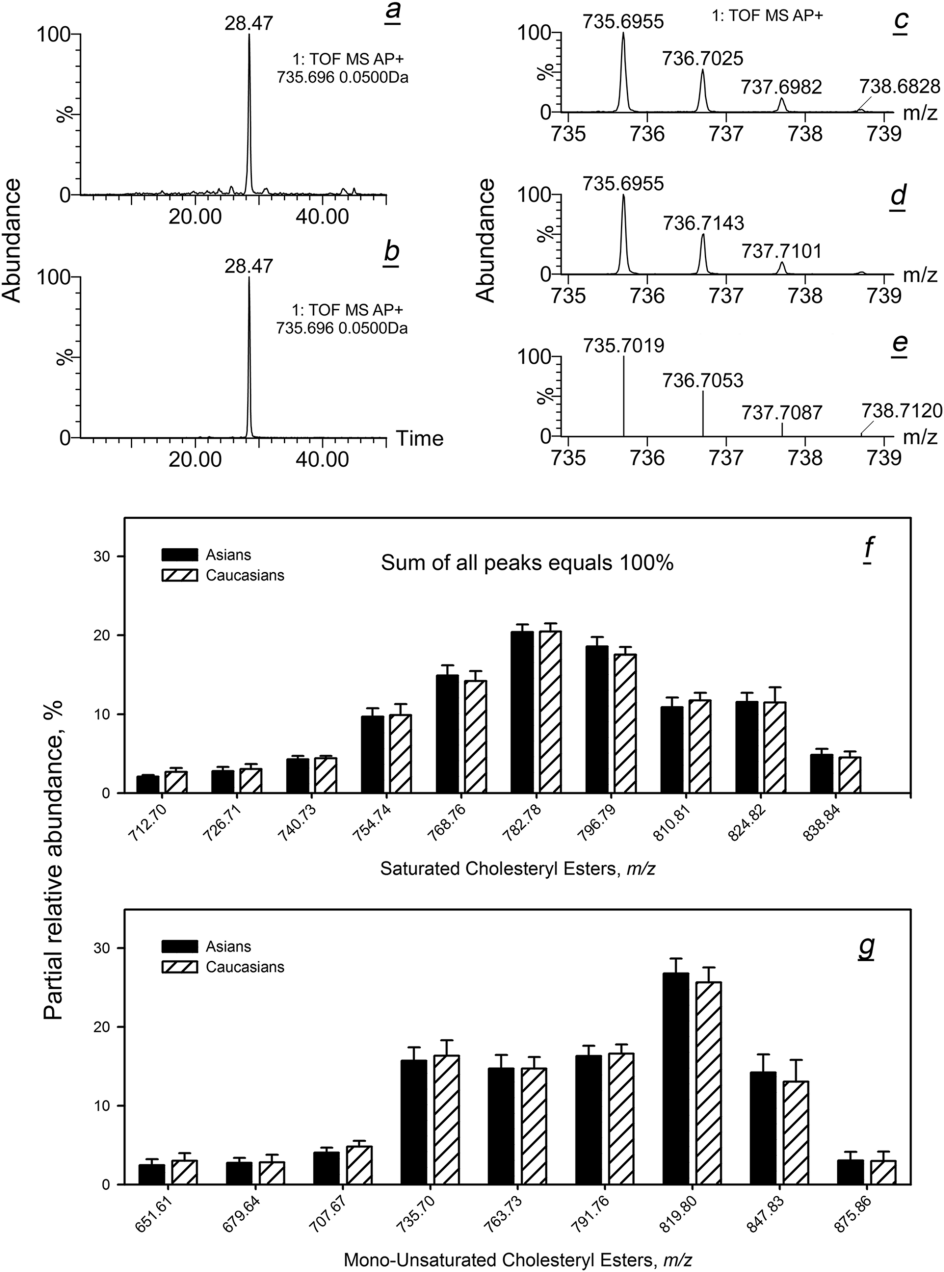
Inter- and intra-group variability of individual cholesteryl esters in Asian and Caucasian population. (**a**) Extracted APCI PIM ion chromatogram (EIC) of meibomian cholesteryl nervonate. (**b**) EIC of authentic cholesteryl nervonate. (**c**) High resolution mass spectrum of the meibomian ester. (**d**) High resolution spectrum of authentic cholesteryl nervonate. (**e**) Theoretical mass spectrum of cholesteryl nervonate. (**f**) Distribution of molecular species of saturated cholesteryl esters in Asian and Caucasian meibum (normalized data). (**g**) Unsaturated cholesteryl esters of Asian and Caucasian meibum.

As an example, EIC of a meibomian C_24:1_-CE with an experimental *m/z* 735.6955, its mass spectrum and a chromatogram and a mass spectrum of authentic cholesteryl nervonate are shown in Fig. 6a–d. The experimental spectra matched a theoretical MS spectrum of its (M + 1) to (M + 4) isotopomers (Fig. 6e). Using *m/z* values from Table S2, EIC of a range of individual CE were obtained, integrated, and compared using their PRA (Fig. 6f,g). Evidently, the profiles of individual meibomian CE were not affected by the ethnicity of the subjects, and the standard deviations were small. Also identical in both ethnicities was the ratio of Chl to total CE: 0.0123 in Asians and 0.0127 in Caucasians.

**Figure 7:**
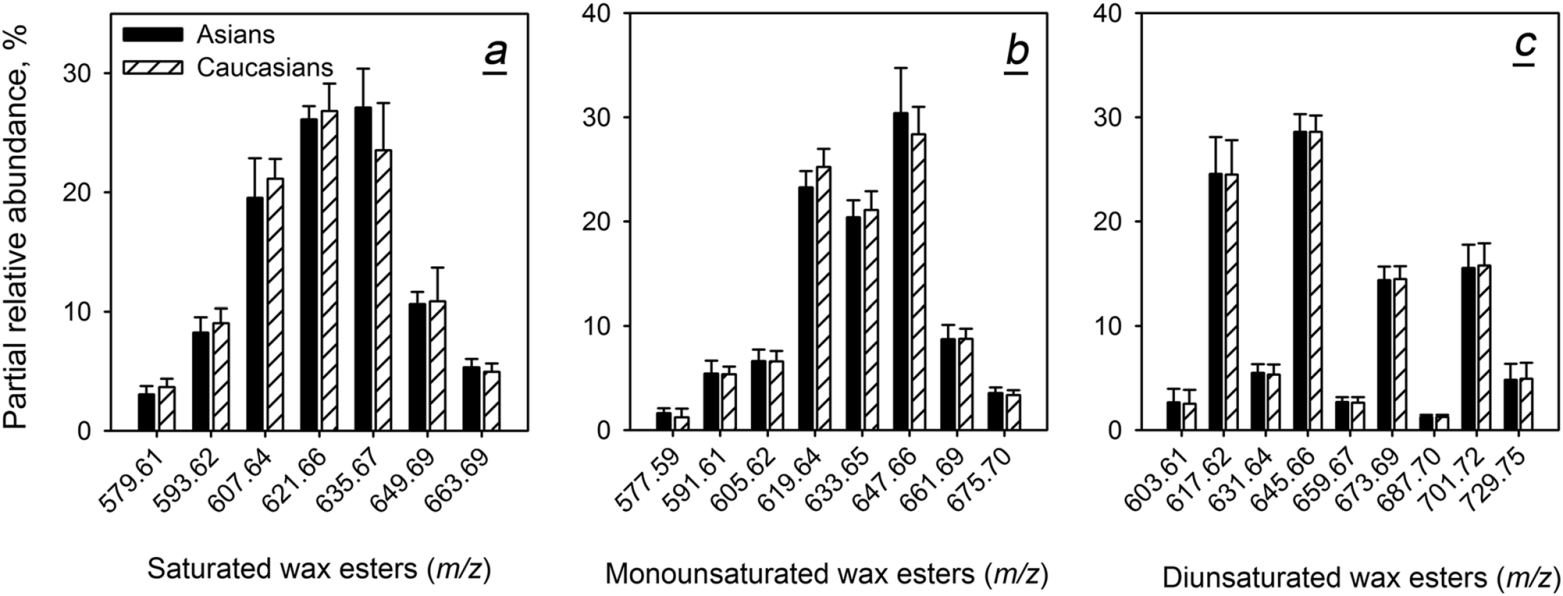
Inter- and intra-group variability of individual wax esters in Asian and Caucasian population. (**a**) Saturated wax esters (normalized). (**b**) Mono-unsaturated wax esters. (**c**) Di-unsaturated wax esters.

Then, major saturated, mono- and di-unsaturated WE were evaluated (Fig. 7). All tested WE were expressed at nearly the same levels in both study groups. Interestingly, saturated WE produced a bell-shaped compound profile with a clear maximum at C_42_H_82_O_2_ and C_43_H_84_O_2_, while the compound profile of mono-unsaturated WE had a reproducible valley at C_43_H_84_O_2_ and two maxima at C_42_H_82_O_2_ and C_44_H_86_O_2_. The makeup of di-unsaturated WE was even more complex, clearly favoring compounds with an even number of carbons C_42_H_80_O_2_, C_44_H_84_O_2_, C_46_H_88_O_2_, and C_48_H_92_O_2_.

**Figure 8:**
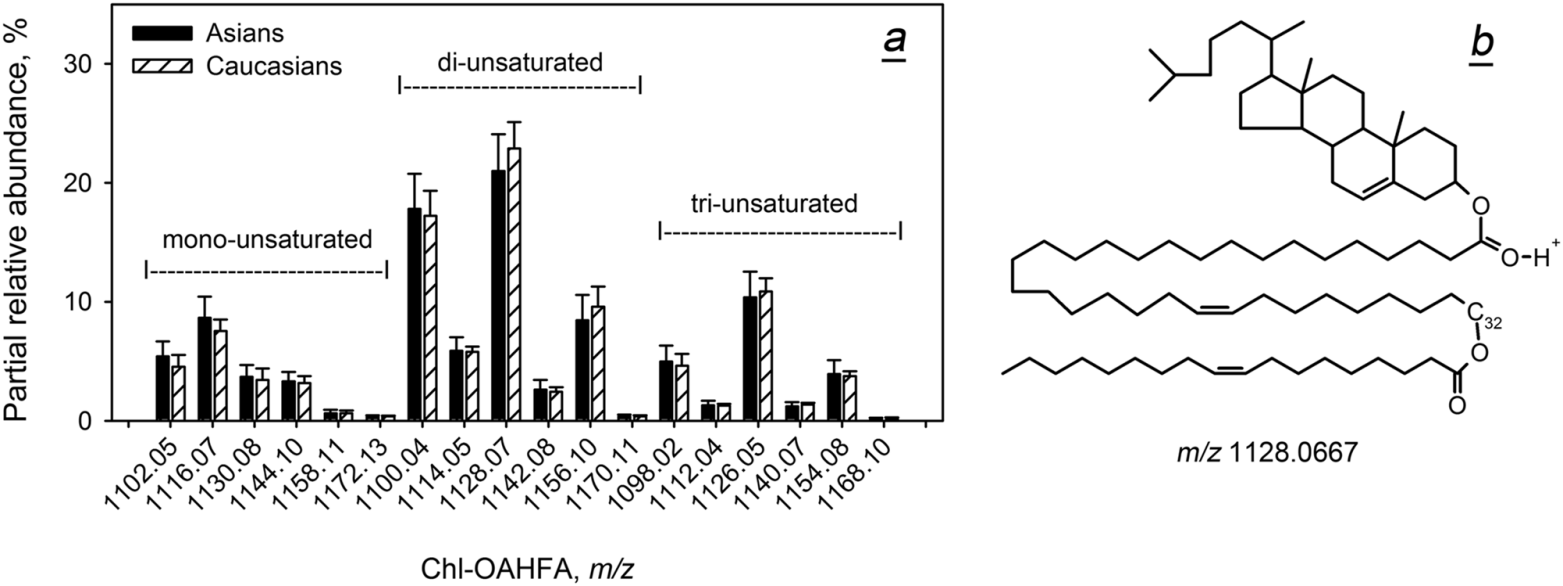
Inter- and intra-group variability of individual cholesteryl esters of *(O)-*acylated ω-hydroxy fatty acids (Chl-OAHFA) in Asian and Caucasian population. (**a**) Distribution of molecular species of Chl-OAHFA (normalized). (**b**) Molecular structure of the major Chl-OAHFA in human meibum.

**Figure 9:**
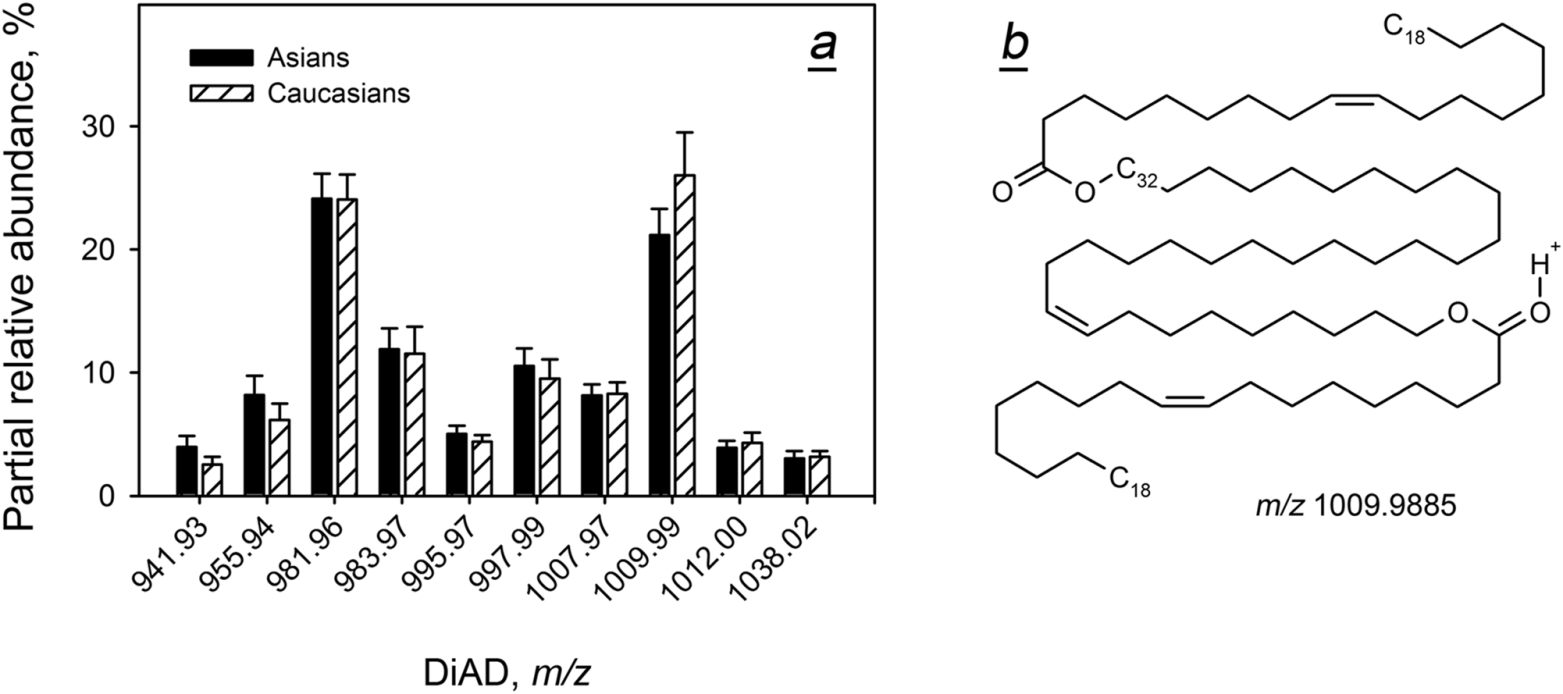
Inter- and intra-group variability of individual cholesteryl esters of di*-*acylated α,ω-diols (DiAD) in Asian and Caucasian population. (**a**) Distribution of molecular species of DiAD (normalized). (**b**) Molecular structure of one of the major DiAD in human meibum.

Next, the distribution of molecular species of major diesters—extremely long chain Chl-OAHFA and DiAD—was investigated (Figs. 8 and 9). For both ethnicities, the pools of Chl-OAHFA were dominated by di-unsaturated compounds with even numbers of carbon atoms in their OAHFA moieties (*m/z* 1,100 and 1,128). As with other tested classes of lipids, the expression levels of Chl-OAHFA were race-independent, and so were the levels of DiAD. Notably, all major DiADs were of di-, tri-, and tetra-unsaturated nature, some of which are shown in Table S2 and Fig. 9b.

**Figure 10:**
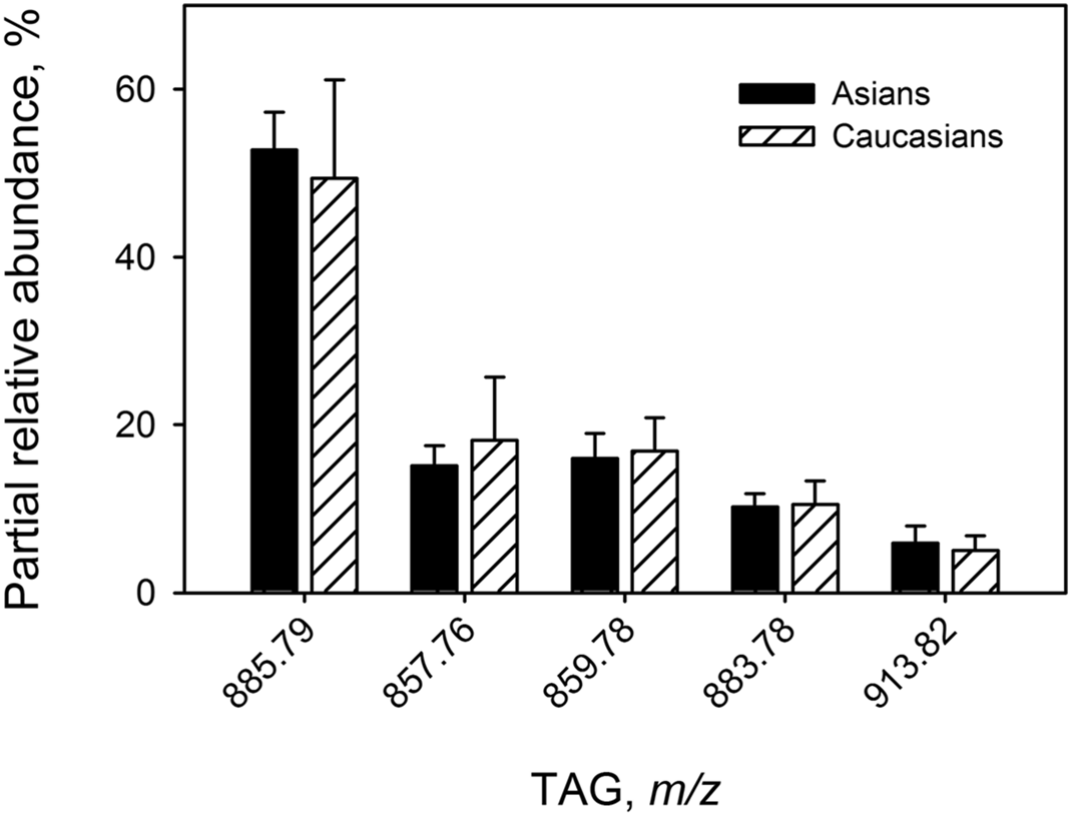
Inter- and intra-group variability of individual cholesteryl esters of triacylglycerols (TAG) in Asian and Caucasian population. Triolein (*m/z* 885.79, detected as a proton adduct) was the main TAG in every sample of human meibum representing close to 40% of the TAG pool.

TAG—a diverse group of nonpolar lipids that mainly fulfill the role of energy and carbon storage—were investigated and found to be identical in both races (Fig. 10). Unlike CE, WE, Chl-OAHFA, and DiAD, all TAG had almost exclusively C_14_ to barely C_22_ fatty acids in all positions: No extremely long chain FA were detected in any of the tested TAG species. The main TAG in all samples was triolein, which accounted for ≥ 40% of the pool.

**Figure 11:**
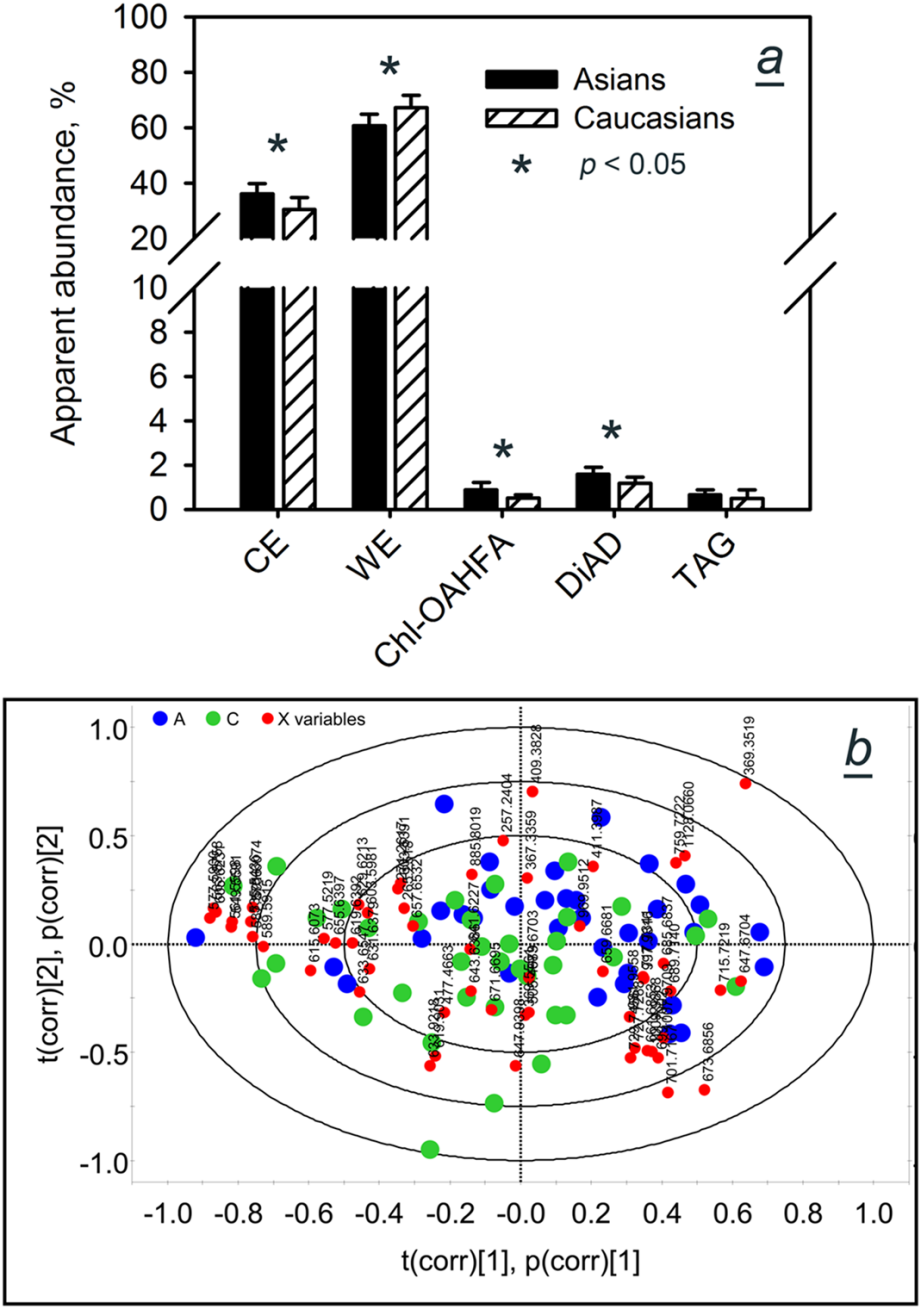
Targeted and untargeted lipidomic analyses of Asian and Caucasian meibum. (**a**) Inter- and intra-group variability of major nonpolar lipid classes in Asian and Caucasian population. CE and WE dominated the meibomian lipidomes in both races and were present at similar levels. Note that apparent abundances are proportional to, but do *not* equate, molecular ratios. (**b**) A PCA biplot of study samples. Scores: Asian samples—blue dots; Caucasian samples—green dots. Loadings: red dots.

A combined graph for tested nonpolar lipids is shown in Fig. 11a. Asian samples were marginally enriched with CE, with a RA of (36 ± 4)% compared to (31 ± 4)% for Caucasians. Though statistically significant (*p* ≤ 0.05), the measured difference was an order of magnitude smaller than one would expect from earlier studies^26^.

**Figure 12:**
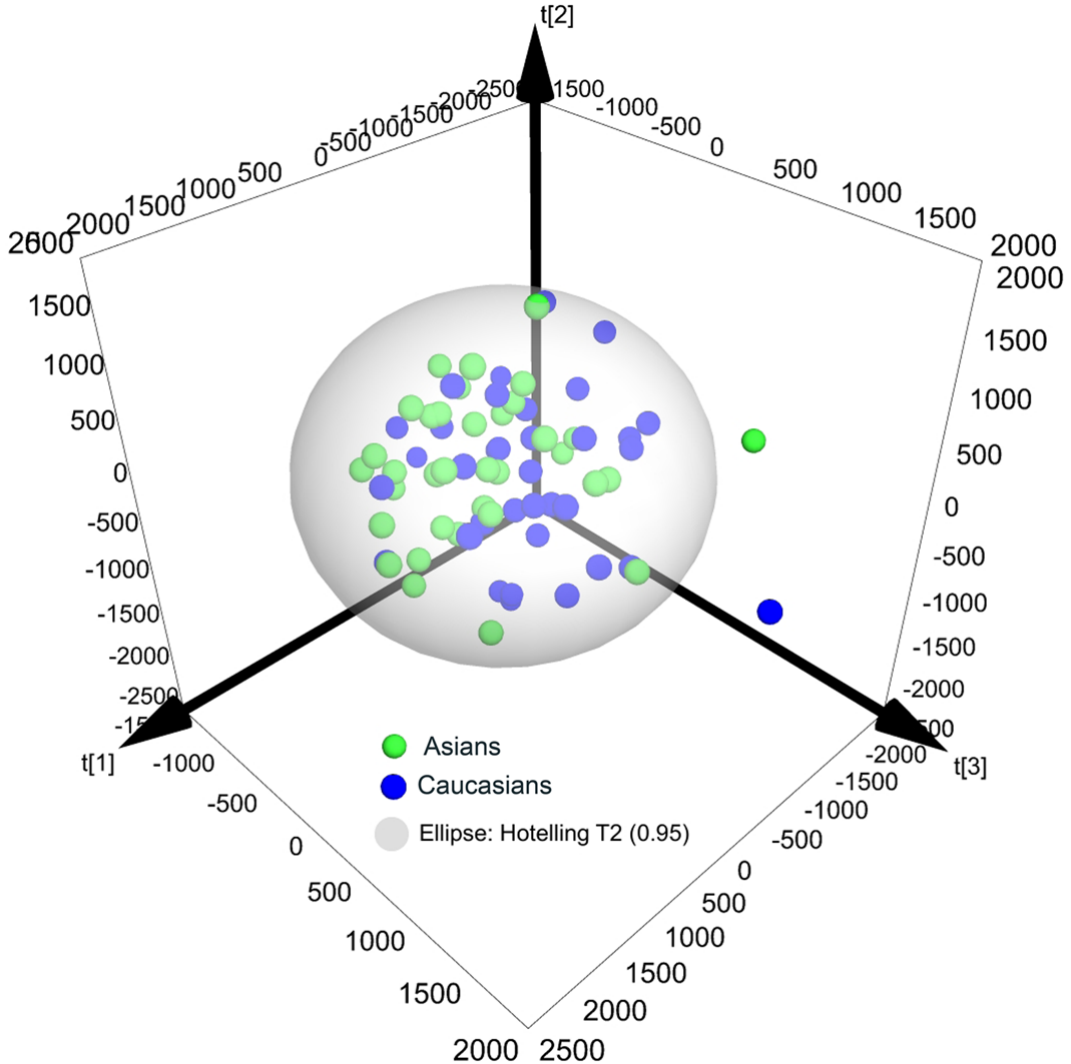
A  3D {PC1 vs. PC2 vs. PC3} scores plot for Asian and Caucasian meibum samples. Note a high degree of overlapping of the samples of two kinds within the Hotelling T2 (0.95) range. Samples that are significantly different from the rest of the pool are located outside the sphere.

Close similarities between Asian and Caucasian meibum were confirmed using the unsupervised Principal Component Analysis (PCA) of the raw RP-UPLC/APCI-MS data files. The model required at least 11 scores to explain 95% of the variances (Supplemental Fig. S1). Thus, only {PC1 vs. PC2}and {PC1 vs. PC2 vs. PC3}graphs are presented in the paper. The PCA biplot for the two types of samples (Fig. 11b) demonstrated two highly overlapping clusters of scores (i.e. samples). A minor separation between Asian and Caucasian samples, and a tighter clustering of the Asian samples in the same two right quadrants, were attributed to the effect of Chl-containing compounds (*m/z* values of 369.3519, 1,128.0660, 1,009.9868 and others) and long chain mono- and di-unsaturated WE (*m/z* 647.6704, 659.6681, 661.6853, 673.6856, 689.7140, 699.7002, 701.7167, 715.7219, and 729.7484), while shorter chain WE (*m/z* 563.5758, 577.5900, 589.5915, 591.6074, 605.6231, 615.6073, 619.6392, 631.6379 and 633.6547) grouped in the left two quadrants. Their chemical assignments are shown in Table S2. However, when three principal components (PC1, PC2 and PC3) are plotted in a 3D graph (Fig. 12), the similarity between Asian and Caucasian meibum became even more evident, with just a few outliers falling beyond the Hotelling T2 ellipse. Note that intra-group sample-to-sample variability for both groups were of the same order of magnitude as the inter-group differences. Thus, supervised approaches are generally considered a better option for highly similar samples^53^.

**Figure 13:**
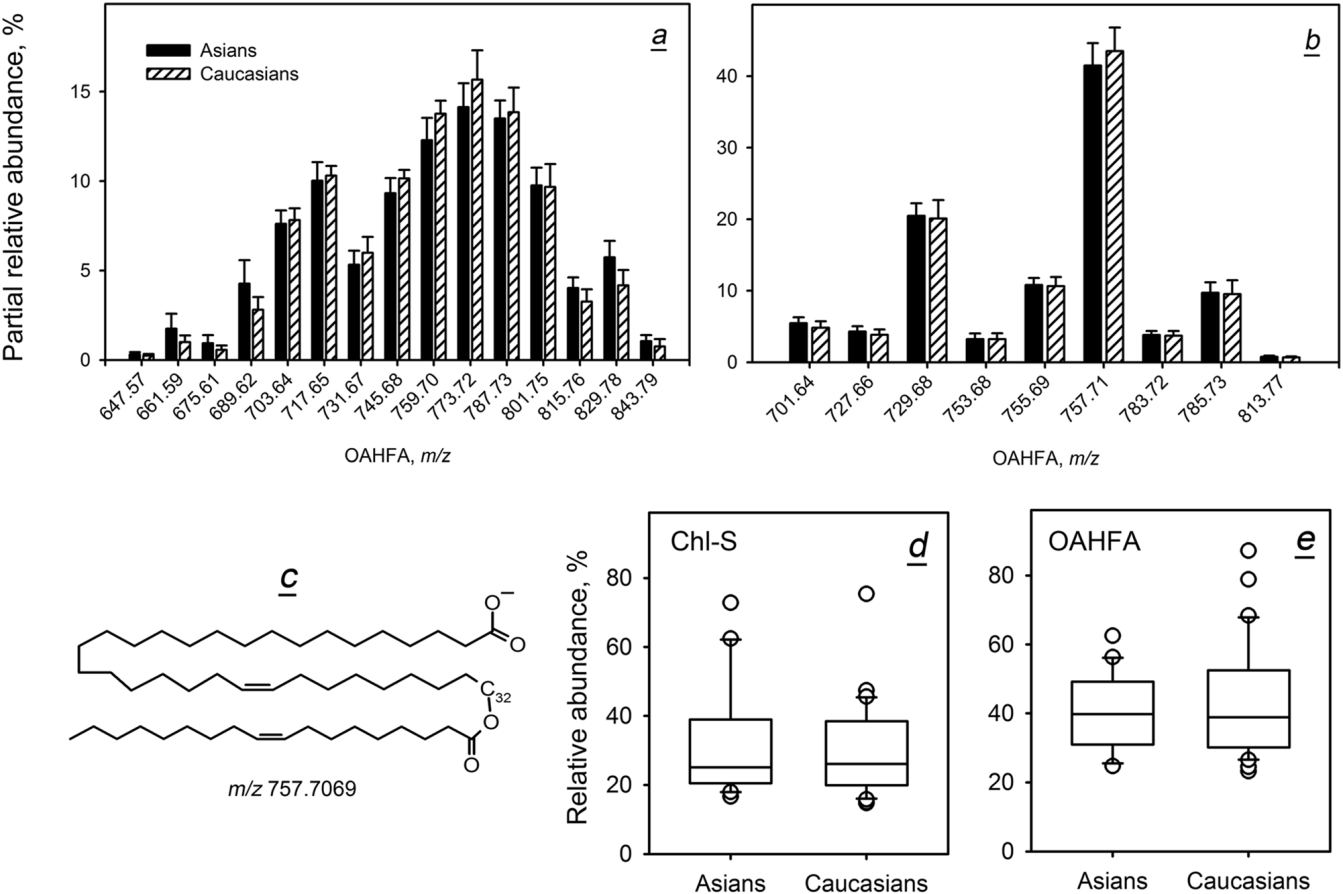
Inter- and intra-group variability of polar lipids in Asian and Caucasian population. (**a**) Distribution of molecular species of mono-unsaturated *(O)-*acylated ω-hydroxy fatty acids (OAHFA) (normalized). (**b**) Distribution of molecular species of di-unsaturated OAHFA. (**c**) Molecular structure of the major OAHFA in human meibum. (**d**) The box plot for the overall presence of cholesteryl sulfate in human meibum. (**e**) The box plot for the overall presence of OAHFA in human meibum. The Mann–Whitney Rank Sum tests confirmed that there were no ethnicity-associated differences in the distribution of Chl-S and OAHFA between races.

Finally, major polar lipids of meibum—OAHFA—were compared using negative ion mode MS. Alongside OAHFA, another interesting compound—cholesteryl sulfate (Chl-S, C_27_H_46_O_4_S, theoretical *m/z* of anion 465.3038)—was also monitored. All OAHFA detected in meibum of both ethnicities (Fig. 13a,b and Table S2) belong to the family of extremely long chain lipids with their mono-unsaturated and di-unsaturated ω-hydroxy FA moieties ranging from C_26_, at least, C_36_ (Fig. 13c). The major acylating FA were of C_16_ and C_18_ nature with one and two double bonds, while a much smaller percentage of OAHFA had tri-unsaturated FA moieties. As with other classes of lipids, there were no differences detected between Asian and Caucasian samples in the molecular distribution of various OAHFA species. To determine if the overall amounts of OAHFA were different or similar in meibum of two races, the OAHFA's RA were calculated using Eq. (1). Their values were found to be almost identical for Asians and Caucasians—40.2% and 43.2%, respectively. However, the standard deviations for both races were rather high (10.8% for Asians and 16.2% for Caucasians), placing OAHFA amongst the most variable groups of meibomian lipids. Similar results were obtained for Chl-S—31.4 ± 16.2% for Asians and 30.1 ± 12.7% (mean ± SD) for Caucasians. To verify these conclusions, standard box plots for both ethnicities were generated (Fig. 12d,e). The Mann–Whitney Rank Sum test confirmed that there were no ethnicity-associated differences in the RA of Chl-S: their median values were 25.1% and 26.0% for Asians and Caucasians, respectively (Mann–Whitney *U* statistic = 319; *p* = 0.911). The same conclusion was made with regard to OAHFA: their median values were 39.9% for Asians and 38.9% for Caucasians (*U* = 310; *p* = 0.78). Note that just a handful of samples fell in the 10th and 90th percentile and could be considered outliers: the vast majority of the samples were in the 25th and 75th percentiles. Thus, irrespective of the implemented analytical techniques, no noticeable differences between Asian and Caucasian OAHFA and Chl-S were observed.

## Discussion

The main goal of this project was to determine to which extent, if any, ethnicity (or race) can influence the lipid composition of meibum, which is the main source of lipids for TF^36^. According to the prevailing view, TF has a multilayered structure composed of an aqueous layer that is covered with the TF lipid layer (TFLL)^54^. The latter is thought to provide a physical barrier for evaporation of the underlying aqueous layer due to the outermost nonpolar lipid sublayer^55,56^, which is formed mostly of highly hydrophobic compounds such as WE, CE, Chl-OAHFA, DiAD, and TAGs. Beneath this nonpolar sublayer lays a much thinner, but critically important, amphiphilic lipid sublayer which is enriched with amphiphiles: Chl, OAHFA, free FA, etc.^30,43^. The role of these amphiphiles is to serve as a stabilizing interface between water-immiscible nonpolar lipids and the aqueous layer which is mostly formed of aqueous tears. Thus, changes in meibum lipids may affect the ability of TF to protect the ocular surface by compromising the thickness and integrity of the nonpolar lipid sublayer, or the ability of the amphiphilic lipids to support it. TF and TFLL are often compromised in patients with DES and MGD^16,57^. Theoretically, differences in the chemical composition of meibum *may* contribute to a higher prevalence of DES and MGD in the Asian population compared to the Caucasian one, as many epidemiologic studies demonstrated^58–60^. To test this hypothesis, a direct comparison of meibum from both ethnic groups was performed in this study.

As a starting point, we chose the original publications by Lam et al.^26,32^ who reported that Asian meibum was highly enriched with CE, which comprised between 67% and 49% of total meibum lipid. Note that those results must not be directly compared with our data as the latter number (49%) was obtained for DES patients of Asian descent with no data on normal controls, while the former estimate (67%) was unreliable because of the deficiencies in experimental techniques discussed earlier^30^. Either of the reported levels of Asian CE was considerably higher than our previous estimates for Caucasians (around 31% of all detectable lipids)^27–30^. The WE levels in Asian samples, per Lam et al., also differed from those in Caucasian ones, accounting for 25%–43% of all lipids (depending on the publication^26,32^) but was about 41% for Caucasians in our hands^33^. Importantly, all of our earlier studies were conducted with predominantly Caucasian volunteers who represented the local population in Texas—a mix of mostly whites, some Hispanics/Latinos, African-Americans, and just a few Asian participants (note a paper by Liu et al. on the topic^61^). The low number of Asian samples made it impossible to account for the role of ethnicity in meibogenesis. At the same time, Lam et al. studied exclusively Asian population with no Caucasian samples analyzed for comparison purposes. Other factors to consider were obvious differences in methodologies implemented in our two laboratories (such as ESI MS in experiments by Lam et al., vs. APCI MS used in our studies), which added another level of uncertainty to the direct comparison of the results. Our current study was designed to overcome these limitations and compare Asian and Caucasian meibum side-by-side in the identical experimental settings using both ESI and APCI techniques.

Previously, we identified and quantitated major meibomian lipids—WE and CE—by LC-APCI MS^n^ and GC/electron impact MS^n^ using a range of homologous chemical standards^28,29,31,37,44,45,62^. Since lineups of standards for quantitation of many remaining classes of lipids (such as Chl-OAHFA, DiAD, and others) were (and still are) unavailable, to compare groups of samples in this paper we relied on the analysis of *intersample* and *intergroup* differences, similar to the analysis of *fold changes*, which is a widely used approach in other disciplines. As PRA of none of the analytes differed between the samples by more than 10–20%, the use of straight fold changes was not practical and percentage points were calculated instead.

Initially, all samples were analyzed by ESI MS to approximate the conditions of the experiments of Lam et al.^26^. The "Combine All Files" routine of the MassLynx software package proved to be a useful tool for the initial characterization of Asian and Caucasian study groups without the need to extract and integrate signals of each of the analytes for each sample separately: resulting two files—ADF-A and ADF-C—already contained averaged information for each analyte. Also, this approach considerably improved signal-to-noise ratio helping to separate minor analytes from background noise. TIC, observation mass spectra, and EIC for selected analytes of both study groups are shown in Figs. 2, 3, and 4. One can see that the averaged chromatograms and spectra were similar in appearance, with only minor differences that were well within the boundaries of previously reported natural variability of meibomian lipids for a given study group—(15 ± 5)%, or less, of the mean values^19,29,51,52^. The unbiased, unsupervised PCA analysis reconfirmed this conclusion as a 3D PC scores plot showed a rather tight grouping of meibum samples regardless the ethnicity (Fig. 12). To explain 95% of variance, 11 principal components were needed (Supplemental Figure S1). Because of the space limitations, only PC1 through PC3 are shown in Figs. 11b and 12. The data in the PCA loadings biplot shown in Fig. 11b demonstrated that some of the Asian samples were slightly pooled toward CE and longer-chain WE, while some of the Caucasian ones were partially associated with shorter chain lipids. However, a substantial overlap of the two groups in the center of the graph provided support of our finding that Asian meibum closely resembles the Caucasian one.

The same conclusions were arrived at when analyzing individual samples separately (Figs. 6, 7, 8, 9, 10, 11 and 13), with a fairly small difference between meibum of Asians and Caucasians being a slightly higher fraction of total CE in the former: the mean value for both races was (33.5 ± 2.5)%. It remains to be seen if a difference of this magnitude—± 7.7% of the mean—can have a physiologically significant impact on TF and TFLL, especially considering highly similar molecular compositions of the pools of WE, CE, and other lipids of meibum.

There are other factors that could explain a large discrepancy between the data of Lam et al.^26^ and our earlier reports on the overall presence of WE in human meibum. Our estimate of about 40%^30^ included a group of saturated WE, which were not detected by Lam et al.^26^, but in our hands represented at least 20% of the total WE pool^33^. In a later report^32^, Lam et al. raised the numbers for total WE from 25 to 43% (which matched our results), but only for DES patients. The major obstacle in detecting saturated WE is their extremely poor, and varying, ionizability in the conditions of LC/MS analyses, and formation of other types of ions (e.g. sodium and ammonium adducts), which leads to a decline in (M + H)^+^ species and complicates interpretation of their mass spectra. When saturated and unsaturated WE standards were analyzed as an equimolar mixture, the loss of ionization efficacy of saturated WE led to a 90–98% lower abundance of their proton adducts, compared to mono-unsaturated WE (data not shown). Also, the effect depended on the molecular weight of WE. These phenomena may impact results of lipidomic analyses of meibum and other types of samples, and will be addressed in detail in future studies.

We also separated regular CE from Chl-OAHFA—a related, but different class of cholesterol-containing compounds^19,21,28,31,44,45,47^, which represented at least 3% of total meibum lipid, or ~ 9% of total pool of Chl-containing lipids, but was not reported and accounted for by Lam et al.. Yet another compound which was not detected by Lam et al. as a separate analyte was free Chl. Per our data, Chl consistently represents at least 0.5% of total meibum, or 1.5% of the CE pool. These factors, if properly accounted for, bring the CE/WE ratio reported by Lam et al. much closer to our earlier estimates than they appear without corrections for saturated WE, Chl, and Chl-OAHFA. Importantly, no gross difference in the CE/WE ratio between Asians and Caucasians was found in an independent study^23^, though the implemented technique—NMR—by its nature is not well-suited for identifying and quantifying any individual lipids in complex mixtures of homologous compounds (such as meibum), and could not differentiate between CE and Chl-OAHFA, WE and DiAD, etc., having most value as a supplementary technique once the chemical composition of samples have been established by other methods.

Amphiphilic OAHFA were proposed to serve as stabilizers of TFLL^30,43–45,62–64^, and their decline in meibum of DES patients was reported by Lam et al.^26^. Thus, it was important to determine if there were any differences in the levels of OAHFA between the two ethnicities: The higher frequency of DES/MGD in Asian population could be related to the lower presence of OAHFA in their meibum compared with Caucasians. However, our experiments demonstrated that the makeup and the overall presence of OAHFA did not differ between the ethnicities (Fig. 13). The levels of another amphiphile—Chl-S—were also identical in Asians and Caucasians. The proposed role of Chl-S—being a part of the amphiphilic lipid sublayer^32^—seems unlikely at this time because of its extreme polarity: Its retention time (less than 2.5 min) in RP-UPLC conditions was the shortest of all tested analytes. It would be important to determine if Chl-S plays any role in the ocular surface physiology, stabilizing or destabilizing TFLL, or it is a catabolic product of Chl and its esters meant to be removed from the ocular surface with tears.

In conclusion, our experiments revealed that composition of major lipid classes: WE, CE, TAG, OAHFA, Chl-OAHFA, and DiAD, remained invariable in both races, barring a slight increase in the Asian CE to WE ratio, which was well within the natural variability ranges for meibomian lipids reported in previous studies. It remains to be seen if a difference of this magnitude could be responsible for higher prevalence of DES/MGD in the Asian population compared to Caucasians. Thus, regardless of the ethnicity, climate, lifestyle, diet, etc., the gross inter-populational differences between normal Asian and Caucasian meibum were deemed to be minimal (Figs. 11 and 12) implying no major differences in meibogenesis in two races in normal conditions.

## Supplementary information

Supplementary information

## Data Availability

All the data that are pertinent to this manuscript are included in the tables and figures. The readers are encouraged to request additional information from the corresponding author.

## Abbreviations

APCI: Atmospheric pressure chemical ionization
CE: Cholesteryl ester(s)
Chl: Free cholesterol
Chl-OAHFA: Cholesteryl ester(s) of *(O)*-acylated ω-hydroxy fatty acid(s)
DES: Dry eye syndrome
DiAD: Diacylated α,ω-diol(s)
EIC: Extracted ion chromatogram
FFA: Free fatty acid(s)
FWHM: Full width at half maximum (resolving power)
MG: Meibomian gland(s)
MGD: Meibomian gland dysfunction
NIM: Negative ion mode
OAHFA: *(O)*-Acylated ω-hydroxy fatty acid(s)
PIM: Positive ion mode
PRA: Partial relative abundance
RA: Relative abundance
RP: Reversed phase
RT: Retention time(s)
TAG: Triacylglycerol(s)
TIC: Total ion current chromatogram(s)
WE: Wax ester(s)
