# Supplementary material for: Comprehensive profiling of Asian and Caucasian meibomian gland secretions reveals similar lipidomic signatures regardless of ethnicity
Source: Sci Rep. 2020 Sep 3;10:14510. doi: 10.1038/s41598-020-71259-5 (PMC7471331; doi:10.1038/s41598-020-71259-5)
Supplement: Supplementary file 1 — Supplementary information [file 41598_2020_71259_MOESM1_ESM.docx]

SUPPLEMENTAL MATERIALS:

**Comprehensive Profiling of Asian and Caucasian Meibomian Gland Secretions**

**Reveal Similar Lipidomic Signatures Regardless of Ethnicity**

Igor A. Butovich^1,2,*^, Tomo Suzuki^3,4^, Jadwiga Wojtowicz^1,#^, Nita Bhat^1^, Seher Yuksel^1^

^1^ Department of Ophthalmology and ^2^Graduate School of Biomedical Sciences, University of Texas Southwestern Medical Center, Dallas, TX, USA

^3^ Department of Ophthalmology, Kyoto Prefectural University of Medicine, Kyoto, Japan

^4^ Department of Ophthalmology, Kyoto City Hospital Organization, Kyoto, Japan

*To whom correspondence should be addressed at: Department of Ophthalmology, University of Texas

Southwestern Medical Center, 5323 Harry Hines Blvd., Dallas, TX 75390-9057, USA

E-mail: igor.butovich@utsouthwestern.edu

**Supplemental Table S1.** Study subjects.

| Race | Asians | | Caucasians | |
| --- | --- | --- | --- | --- |
| Sex | Males | Females | Males | Females |
| N | 17* | 18 | 11 | 25** |
| Age range, ys | 27-70 | 20-69 | 29-75 | 28-72 |
| Average age, ys | 49 | 45 | 54 | 51 |
| SD | 17 | 20.0 | 18 | 13 |

* One study sample of 18 in total was disqualified because of unfavorable signal-to-noise ratios.

**Two study samples from 27 in total were disqualified.

**Supplemental Table S2.** Lipid analytes.

| Lipid | Molecular formula | Major ion species | Theoretical  *m/z* | Structure and  main isobaric isomers | | Other characteristic ions in MS and MS^E^ |
| --- | --- | --- | --- | --- | --- | --- |
| **Sterols** | | | | | | |
| Cholesterol (free) | C_27_H_46_O | (M – H_2_O + H)^+^ | 369.3521 |  | |  |
| Cholesteryl sulfate | C_27_H_46_O_4_S | (M – H)^−^ | 465.3038 |  | |  |
| **Cholesteryl esters**  Fatty acid(=O)-O-Cholesterol | | | | | | |
| Saturated | C_48_H_86_O_2_ | (M + NH_4_)^+^ | 712.6971 | C_21:0_(=O)-O-Chl | | (M + Na)^+^ and  (M + H)^+^ (minor) |
|  | C_49_H_88_O_2_ |  | 726.7128 | C_22:0_(=O)-O-Chl | |  |
|  | C_50_H_90_O_2_ |  | 740.7284 | C_23:0_(=O)-O-Chl | |  |
|  | C_51_H_92_O_2_ |  | 754.7437 | C_24:0_(=O)-O-Chl | |  |
|  | C_52_H_94_O_2_ |  | 768.7597 | C_25:0_(=O)-O-Chl | |  |
|  | C_53_H_96_O_2_ |  | 782.7754 | C_26:0_(=O)-O-Chl | |  |
|  | C_54_H_98_O_2_ |  | 796.7910 | C_27:0_(=O)-O-Chl | |  |
|  | C_55_H_100_O_2_ |  | 810.8067 | C_28:0_(=O)-O-Chl | |  |
|  | C_56_H_102_O_2_ |  | 824.8223 | C_29:0_(=O)-O-Chl | |  |
|  | C_57_H_104_O_2_ |  | 838.8380 | C_30:0_(=O)-O-Chl | |  |
|  | C_58_H_106_O_2_ |  | 852.8536 | C_31:0_(=O)-O-Chl | |  |
| Mono-unsaturated | C_45_H_78_O_2_ | (M + H)^+^ | 651.6079 | C_18:1_(=O)-O-Chl | | (M + NH_4_)^+^  (minor) |
|  | C_47_H_82_O_2_ |  | 679.6393 | C_20:1_(=O)-O-Chl | |  |
|  | C_49_H_86_O_2_ |  | 707.6706 | C_22:1_(=O)-O-Chl | |  |
|  | C_51_H_90_O_2_ |  | 735.7019 | C_24:1_(=O)-O-Chl | |  |
|  | C_53_H_94_O_2_ |  | 763.7332 | C_26:1_(=O)-O-Chl | |  |
|  | C_55_H_98_O_2_ |  | 791.7645 | C_28:1_(=O)-O-Chl | |  |
|  | C_57_H_102_O_2_ |  | 819.7958 | C_30:1_(=O)-O-Chl | |  |
|  | C_59_H_106_O_2_ |  | 847.8271 | C_32:1_(=O)-O-Chl | |  |
|  | C_61_H_110_O_2_ |  | 875.8584 | C_34:1_(=O)-O-Chl | |  |
| **Wax esters**  Fatty acid(=O)-O-Fatty alcohol  (mixture of many isobaric isomers) | | | | | | |
| Saturated | C_39_H_78_O_2_ | (M + H)^+^ | 579.6080 | C_17:0_(=O)-O-C_22:0_ | | (FA + H)^+^  (FA – H_2_O + H)^+^  (FA – 2H_2_O + H)^+^  (M + NH_4_)^+^ in ESI |
|  | C_40_H_80_O_2_ |  | 593.6236 | C_17:0_(=O)-O-C_23:0_ | |  |
|  | C_41_H_82_O_2_ |  | 607.6393 | C_16:0_(=O)-O-C_25:0_  C_17:0_(=O)-O-C_24:0_ | |  |
|  | C_42_H_84_O_2_ |  | 621.6549 | C_16:0_(=O)-O-C_26:0_  C_17:0_(=O)-O-C_25:0_  C_18:0_(=O)-O-C_24:0_ | |  |
|  | C_43_H_86_O_2_ |  | 635.6706 | C_17:0_(=O)-O-C_26:0_  C_18:0_(=O)-O-C_25:0_ | |  |
|  | C_44_H_88_O_2_ |  | 649.6862 | C_17:0_(=O)-O-C_27:0_  C_18:0_(=O)-O-C_26:0_ | |  |
|  | C_45_H_90_O_2_ |  | 663.7019 | C_17:0_(=O)-O-C_28:0_  C_18:0_(=O)-O-C_27:0_ | |  |
| Mono-unsaturated | C_39_H_76_O_2_ |  | 577.5920 | C_16:1_(=O)-O-C_23:0_  C_18:1_(=O)-O-C_21:0_ | |  |
|  | C_40_H_78_O_2_ |  | 591.6076 | C_16:1_(=O)-O-C_24:0_  C_18:1_(=O)-O-C_22:0_ | |  |
|  | C_41_H_80_O_2_ |  | 605.6233 | C_16:1_(=O)-O-C_25:0_  C_17:0_(=O)-O-C_24:1_  C_18:1_(=O)-O-C_23:0_ | |  |
|  | C_42_H_82_O_2_ |  | 619.6389 | C_16:1_(=O)-O-C_26:0_  C_18:1_(=O)-O-C_24:0_ | |  |
|  | C_43_H_84_O_2_ |  | 633.6545 | C_16:1_(=O)-O-C_27:0_  C_17:0_(=O)-O-C_26:1_  C_18:1_(=O)-O-C_25:0_ | |  |
|  | C_44_H_86_O_2_ |  | 647.6702 | C_16:1_(=O)-O-C_28:0_  C_18:1_(=O)-O-C_26:0_ | |  |
|  | C_45_H_88_O_2_ |  | 661.6858 | C_17:0_(=O)-O-C_28:1_  C_18:1_(=O)-O-C_27:0_ | |  |
|  | C_46_H_90_O_2_ |  | 675.7015 | C_16:1_(=O)-O-C_30:1_  C_18:1_(=O)-O-C_28:0_ | |  |
| Di-unsaturated | C_41_H_78_O_2_ |  | 603.6080 | C_18:2_(=O)-O-C_23:0_  C_18:1_(=O)-O-C_23:1_ | |  |
|  | C_42_H_80_O_2_ |  | 617.6236 | C_16:1_(=O)-O-C_26:1_  C_18:1_(=O)-O-C_24:1_  C_18:2_(=O)-O-C_24:0_ | |  |
|  | C_43_H_82_O_2_ |  | 631.6393 | C_17:0_(=O)-O-C_26:2_  C_18:2_(=O)-O-C_25:0_ | |  |
|  | C_44_H_84_O_2_ |  | 645.6549 | C_18:1_(=O)-O-C_26:1_ | |  |
|  | C_45_H_86_O_2_ |  | 659.6706 | C_17:0_(=O)-O-C_28:2_ | |  |
|  | C_46_H_88_O_2_ |  | 673.6862 | C_18:1_(=O)-O-C_28:1_ | |  |
|  | C_47_H_90_O_2_ |  | 687.7019 | C_17:0_(=O)-O-C_30:2_ | |  |
|  | C_48_H_92_O_2_ |  | 701.7175 | C_18:1_(=O)-O-C_30:1_ | |  |
|  | C_49_H_94_O_2_ |  | 715.7327 | C_18:2_(=O)-O-C_31:0_ | |  |
|  | C_50_H_96_O_2_ |  | 729.7488 | C_18:1_(=O)-O-C_32:1_ | |  |
| **Chl-OAHFA**  Fatty acid(=O)-[O-ω-Fatty acid(=O)]-O-cholesterol  (mixture of many isobaric isomers) | | | | | | |
| Mono-unsaturated | C_75_H_136_O_4_ | (M + H)^+^ | 1102.0516 | C_18:1_(=O)-O-C_30:0_(=O)-O-Chl | | (M – Chl + H)^+^  (M – H_2_O -Chl + H)^+^  (Chl – H_2_O + H)^+^ |
|  | C_76_H_138_O_4_ |  | 1116.0673 | C_18:1_(=O)-O-C_31:0_(=O)-O-Chl | |  |
|  | C_77_H_140_O_4_ |  | 1130.0829 | C_18:1_(=O)-O-C_32:0_(=O)-O-Chl | |  |
|  | C_78_H_142_O_4_ |  | 1144.0986 | C_18:1_(=O)-O-C_33:0_(=O)-O-Chl | |  |
|  | C_79_H_144_O_4_ |  | 1158.1142 | C_18:1_(=O)-O-C_34:0_(=O)-O-Chl | |  |
|  | C_80_H_146_O_4_ |  | 1172.1299 | C_18:1_(=O)-O-C_35:0_(=O)-O-Chl | |  |
| Di-unsaturated | C_75_H_134_O_4_ |  | 1100.0360 | C_18:1_(=O)-O-C_30:1_(=O)-O-Chl | |  |
|  | C_76_H_136_O_4_ |  | 1114.0516 | C_18:2_(=O)-O-C_31:0_(=O)-O-Chl | |  |
|  | C_77_H_138_O_4_ |  | 1128.0673 | C_18:1_(=O)-O-C_32:1_(=O)-O-Chl | |  |
|  | C_78_H_140_O_4_ |  | 1142.0829 | C_18:2_(=O)-O-C_33:0_(=O)-O-Chl | |  |
|  | C_79_H_142_O_4_ |  | 1156.0986 | C_18:1_(=O)-O-C_34:1_(=O)-O-Chl | |  |
|  | C_80_H_144_O_4_ |  | 1170.1142 | C_18:2_(=O)-O-C_35:0_(=O)-O-Chl | |  |
| Tri-unsaturated | C_75_H_132_O_4_ |  | 1098.0203 | C_18:1_(=O)-O-C_30:2_(=O)-O-Chl | |  |
|  | C_76_H_134_O_4_ |  | 1112.0360 | C_18:3_(=O)-O-C_31:0_(=O)-O-Chl | |  |
|  | C_77_H_136_O_4_ |  | 1126.0516 | C_18:1_(=O)-O-C_32:2_(=O)-O-Chl | |  |
|  | C_78_H_138_O_4_ |  | 1140.0672 | C_18:3_(=O)-O-C_33:0_(=O)-O-Chl | |  |
|  | C_79_H_140_O_4_ |  | 1154.0829 | C_18:1_(=O)-O-C_34:2_(=O)-O-Chl | |  |
|  | C_80_H_142_O_4_ |  | 1168.0986 | C_18:3_(=O)-O-C_35:0_(=O)-O-Chl | |  |
| **DiAD**  Fatty acid(=O)-[(O-α,ω-Diol-O)]-(O=)Fatty acid  (mixture of many isobaric isomers) | | | | | | |
| Di-unsaturated | C_63_H_120_O_4_ | (M + H)^+^ | 941.92614 | C_18:1_(=O)-O-C_27:0_-O-(=O)C_18:1_ | | (M – FA + H)^+^ |
| Di-unsaturated | C_64_H_122_O_4_ |  | 955.9421 | C_18:1_(=O)-O-C_28:0_-O-(=O)C_18:1_ | |  |
| Tri-unsaturated | C_66_H_124_O_4_ |  | 981.9577 | C_18:1_(=O)-O-C_30:1_-O-(=O)C_18:1_ | |  |
| Di-unsaturated | C_66_H_126_O_4_ |  | 983.9734 | C_18:1_(=O)-O-C_30:0_-O-(=O)C_18:1_ | |  |
| Tri-unsaturated | C_67_H_126_O_4_ |  | 995.9734 | C_18:1_(=O)-O-C_30:1_-O-(=O)C_18:1_ | |  |
| Di-unsaturated | C_67_H_128_O_4_ |  | 997.9890 | C_18:1_(=O)-O-C_30:0_-O-(=O)C_18:1_ | |  |
| Tetra-unsaturated | C_68_H_126_O_4_ |  | 1007.9734 | C_18:1_(=O)-O-C_32:2_-O-(=O)C_18:1_ | |  |
| Tri-unsaturated | C_68_H_128_O_4_ |  | 1009.9890 | C_18:1_(=O)-O-C_32:1_-O-(=O)C_18:1_ | |  |
| Di-unsaturated | C_68_H_130_O_4_ |  | 1012.0047 | C_18:1_(=O)-O-C_32:0_-O-(=O)C_18:1_ | |  |
| Tri-unsaturated | C_70_H_132_O_4_ |  | 1038.0203 | C_18:1_(=O)-O-C_34:1_-O-(=O)C_18:1_ | |  |
| **TAG**  (FA1,FA2,FA3)-Glycerol  (mixture of many isobaric isomers) | | | | | | |
| Tri-unsaturated | C_55_H_100_O_6_ | (M + H)^+^ | 857.7598 | C_16:1_,C_16:1_,C_18:1_-Gly | | (FA – H_2_O)^+^  (FA – 2H_2_O)^+^  (M – FA + H)^+^ |
| Di-unsaturated | C_55_H_102_O_6_ |  | 859.7754 | C_16:1_,C_18:1_,C_16:0_-Gly | |  |
| Tetra-unsaturated | C_57_H_102_O_6_ |  | 883.7754 | C_18:1_,C_18:1_,C_18:2_-Gly | |  |
| Tri-unstaurated | C_57_H_104_O_6_ |  | 885.7911 | C_18:1_,C_18:1_,C_18:1_-Gly | |  |
| Tri-unsaturated | C_59_H_108_O_6_ |  | 913.8224 | C_18:1_,C_18:1_,C_20:1_-Gly | |  |
| **OAHFA**  Fatty acid(=O)-[O-ω-Fatty acid]  (mixture of many isobaric isomers) | | | | | | |
| Mono-unsaturated | C_42_H_80_O_4_ | (M – H)^+^ | 647.5975 | C_18:0_(=O)-O-C_24:0_(=O)OH | (FA – H)^−^  (FA – H_2_O – H)^−^  (HO-ωFA – H)^−^  (HO-ωFA – H_2_O – H)^−^ | |
|  | C_43_H_82_O_4_ |  | 661.6131 | C_18:0_(=O)-O-C_25:0_(=O)OH |  |  |
|  | C_44_H_84_O_4_ |  | 675.6287 | C_18:0_(=O)-O-C_26:0_(=O)OH |  |  |
|  | C_45_H_86_O_4_ |  | 689.6444 | C_18:0_(=O)-O-C_27:0_(=O)OH |  |  |
|  | C_46_H_88_O_4_ |  | 703.6600 | C_18:0_(=O)-O-C_28:0_(=O)OH |  |  |
|  | C_47_H_90_O_4_ |  | 717.6757 | C_18:0_(=O)-O-C_29:0_(=O)OH |  |  |
|  | C_48_H_92_O_4_ |  | 731.6913 | C_18:0_(=O)-O-C_30:0_(=O)OH |  |  |
|  | C_49_H_94_O_4_ |  | 745.7069 | C_18:0_(=O)-O-C_31:0_(=O)OH |  |  |
|  | C_50_H_96_O_4_ |  | 759.7226 | C_18:0_(=O)-O-C_32:0_(=O)OH |  |  |
|  | C_51_H_98_O_4_ |  | 773.7382 | C_18:0_(=O)-O-C_33:0_(=O)OH |  |  |
|  | C_52_H_100_O_4_ |  | 787.7539 | C_18:0_(=O)-O-C_34:0_(=O)OH |  |  |
|  | C_53_H_102_O_4_ |  | 801.7695 | Not determined |  |  |
|  | C_54_H_104_O_4_ |  | 815.7851 |  |  |  |
|  | C_55_H_106_O_4_ |  | 829.8008 |  |  |  |
|  | C_56_H_108_O_4_ |  | 843.8164 |  |  |  |
| Di-unsaturated | C_46_H_86_O_4_ |  | 701.6448 | C_18:1_(=O)-O-C_28:1_(=O)OH |  |  |
|  | C_48_H_90_O_4_ |  | 729.6760 | C_18:1_(=O)-O-C_30:1_-(=O)OH |  |  |
|  | C_50_H_94_O_4_ |  | 757.7073 | C_18:1_(=O)-O-C_32:1_(=O)OH |  |  |
|  | C_52_H_98_O_4_ |  | 785.7386 | C_18:1_(=O)-O-C_34:1_(=O)OH |  |  |
|  | C_54_H_102_O_4_ |  | 813.7699 | C_18:1_(=O)-O-C_36:1_(=O)OH |  |  |
| Tri-unsaturated | C_48_H_88_O_4_ |  | 727.6604 | C_18:1_(=O)-O-C_30:2_(=O)OH |  |  |
|  | C_50_H_92_O_4_ |  | 755.6917 | C_18:1_(=O)-O-C_32:2_(=O)OH |  |  |
|  | C_52_H_96_O_4_ |  | 783.7230 | C_18:1_(=O)-O-C_34:2_(=O)OH |  |  |
| Tetra-unsaturated | C_50_H_90_O_4_ |  | 753.6761 | C_18:2_(=O)-O-C_32:2_(=O)OH |  |  |

**
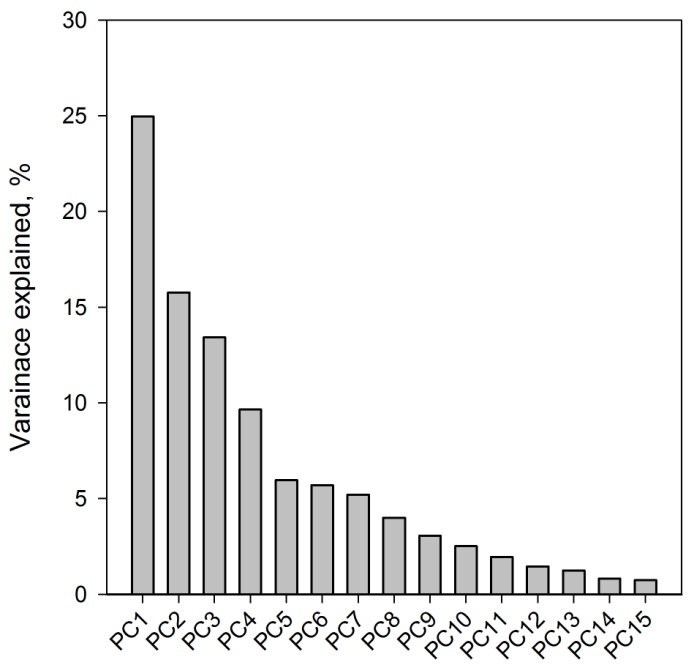
**

**Supplemental Figure S1.** Effects of principal components PC1-PC15 on the variance explained.
